# *Lactobacillus yoelii* Lac-2 mediates the mechanism of MAPK signaling pathway involved in the regulation of intestinal barrier and pathogen translocation by Zonulin expression

**DOI:** 10.1038/s41598-026-55430-y

**Published:** 2026-06-03

**Authors:** Zhongyan Liu, Ziqin Zhang, Meixue Ni, Xiaoli Ren, Xiaoqing Guo, Zhenyu Chang, Hailong Dong, Qingxia Wu

**Affiliations:** 1 Key Laboratory of Clinical Veterinary Medicine, College of Animal Science, Xizang Agricultural and Animal Husbandry University, Linzhi, 860000 People’s Republic of China; 2 Key Laboratory for Prevention and Control of Hydatid Disease in Xizang (Co-constructed by Ministry and Province), Ministry of Agriculture and Rural Affairs, College of Animal Science, Xizang Agricultural and Animal Husbandry University, Linzhi, 860000 Xizang People’s Republic of China

**Keywords:** Yak, Probiotics, Intestinal barrier, MAPK pathway, Zonulin, Mechanistic researchs, Diseases, Gastroenterology, Microbiology, Molecular biology

## Abstract

Yak is an endemic livestock on the Qinghai-Tibet Plateau, which is prone to diarrhea caused by intestinal pathogenic bacteria. In the context of insufficient vaccines and antibiotic resistance, screening lactic acid bacterial strains adapted to high-altitude environments has become a novel approach for the prevention and treatment of diarrhea. This study explored the mechanism by which *Lactobacillus yoelii* Lac-2 regulates the intestinal barrier and pathogen translocation via the MAPK pathway, with a focus on zonulin expression. Zonulin up-regulation and knockdown were performed in intestinal epithelial cell lines, and a monolayer epithelial barrier was established using Transwell chambers. The cells were divided into four groups: control group, *Escherichia coli (E. coli) O78* model group, low- and high-dose *Lactobacillus yoelii* Lac-2 groups. MAPK agonist and inhibitor were added to the corresponding groups, respectively. The expressions of zonulin, mucins (MUC1, MUC2), and tight junction proteins (ZO-1, Occludin, Claudin 1) were detected by RT-qPCR and Western blot. The barrier integrity was evaluated by transepithelial electrical resistance (TEER), FITC-dextran (FD4) permeability, and flow cytometry to detect bacterial translocation and cell apoptosis levels. The results confirmed that zonulin up-regulation and knockdown cell lines were successfully constructed, and the MAPK pathway was effectively intervened. Zonulin upregulation and MAPK pathway activation significantly decreased TEER, increased FD4 permeability and bacterial translocation, downregulated the expressions of MUC1, MUC2, ZO-1, Occludin, and Claudin 1, and promoted cell apoptosis, thereby impairing the intestinal barrier protective effect of *Lactobacillus yoelii* Lac-2. Collectively, yak-derived *Lactobacillus yoelii* Lac-2 can reduce zonulin expression by inhibiting the MAPK signaling pathway, maintain the tight junction structure, and decrease pathogenic bacterial translocation, thereby alleviating *E. coli O78*-induced intestinal epithelial barrier damage and exerting a protective effect on the intestinal barrier.

**Supplementary Information:**

The online version contains supplementary material available at 10.1038/s41598-026-55430-y.

## Introduction

The yak (Bos grunniens) is an endemic livestock species uniquely adapted to the high-altitude Tibetan Plateau (≥ 3000 m), serving as a critical provider of meat, fur, and fertilizer resources for local herders while supporting plateau grassland ecological balance through grazing management^1–5^. However, the sustainable development of the yak industry is increasingly threatened by infectious diseases, with bacterial diarrhea—predominantly caused by pathogenic Escherichia coli (e.g., O78)—emerging as a major challenge. This condition severely impairs calf growth performance, disrupts intestinal function, and causes substantial economic losses, exacerbated by the lack of effective vaccines and the global antibiotic resistance crisis^2,6–8^. These limitations highlight an urgent need for safe, alternative strategies for preventing and treating diarrheal diseases in yaks.

Lactic acid bacteria (LAB), particularly host-derived strains, have gained attention as promising candidates for regulating intestinal health and mitigating bacterial diarrhea, owing to their ability to antagonize pathogens and modulate host immunity^7,9–11^. However, commercially available LAB products often exhibit poor adaptability to the high-altitude hypoxic environment, limiting their efficacy in yaks. To address this gap, our previous work screened and identified Lactobacillus johnsonii Lac-2, a yak-derived strain with robust high-altitude adaptability, and preliminarily demonstrated its protective effects against E. coli O78-induced intestinal barrier damage, potentially via regulating the Zonulin-mediated intestinal permeability and the MAPK signaling pathway^12^. Despite these advances, the precise molecular mechanism by which Lac-2 modulates the interplay between Zonulin expression, the MAPK cascade, and pathogen translocation in yak intestinal epithelial cells remains poorly defined, hindering its translational application as a targeted probiotic intervention.

The intestinal barrier is a multi-layered defense system comprising the mucus barrier (MUC1, MUC2), epithelial cells, tight junction (TJ) proteins (occludin, claudins, ZO-1, etc.), and the gut microbiota, whose integrity is essential for preventing pathogen invasion and systemic inflammation^13–20^. Disruption of TJ proteins, upregulation of the permeability regulator Zonulin, and dysregulation of the mitogen-activated protein kinase (MAPK) signaling pathway—including ERK, JNK, and p38 cascades—are key pathological events in E. coli-induced intestinal injury, as these pathways govern epithelial barrier function, inflammation, and cell turnover^21–25^.

The present study aims to elucidate the molecular mechanism underlying the protective effects of Lactobacillus *yoelii* Lac-2 against *E. coli* O78-induced intestinal barrier dysfunction. To this end, we will construct an in vitro yak intestinal epithelial monolayer barrier model using Transwell inserts, combined with Zonulin overexpression/knockdown and MAPK pathway activator/inhibitor treatments. By analyzing the expression of Zonulin, mucins, and tight junction proteins via RT-qPCR and Western blot, along with assessments of transepithelial electrical resistance (TEER), fluorescein FITC-dextran (FD4) permeability, flow cytometry, and bacterial translocation assays, we seek to define how Lac-2 modulates the MAPK/Zonulin signaling axis to maintain epithelial barrier integrity and inhibit pathogen translocation. These findings will fill critical knowledge gaps in our understanding of host-probiotic-pathogen interactions in high-altitude yaks, and provide a theoretical basis for developing targeted probiotic-based strategies to prevent and treat bacterial diarrhea in this species.

## Materials and methods

### Source of strains

Resuscitation of pathogenic *E.coli* O78 strains of yak origin: Pathogenic *E.coli* O78 was stored in a -80 °C ultra-low temperature freezer with 25% glycerol, and after the cryopreserved bacterial solution was recovered, the strain was detected with eosin-methylene blue agar, and the desired strain concentration was determined by colony-forming unit (CFU) counting.

Yak-derived *Lactobacillus yoelii* Lac-2 resuscitation: *Lactobacillus yoelii* Lac-2 from yak was stored in a -80 °C ultra-low temperature freezer with 25% glycerol, and the strain concentration was determined by CFU counting after the cryopreserved bacterial solution was recovered.

All experimental protocols, including the procurement of yak intestinal epithelial cells, were reviewed and approved by the Institutional Animal Care and Use Committee (IACUC) of Tibet Agricultural and Animal Husbandry University. All procedures were conducted in strict accordance with institutional guidelines, national regulations, and the ARRIVE (Animal Research: Reporting of In Vivo Experiments) Guidelines 2.0. This manuscript is reported in compliance with the ARRIVE Guidelines to ensure transparency and reproducibility.

### Cell culture and reagents

Yak intestinal epithelial cells were isolated and cultured according to the cell culture methods established in our laboratory^26^. The main reagents and materials used in this study were as follows: Eosin blue agar (EMB) (HB0107, Haibo, Qingdao, China), nutrient broth (NB) (HB0108, Haibo, Qingdao, China), MRS medium (HB0384-5, Haibo, Qingdao, China), DMEM-F12 medium (Invitrogen, CA, USA), pancreatic enzyme (containing EDTA) (25200072, Gibco, USA), fetal bovine serum (PAN, Adenbach, Germany), MAPK Activator (A1895-5G, Merck, Germany), MAPK Blocker (SC68376, SCBT, Shanghai, China), RIPA lysis buffer (BL504A, LABGIC, Beijing, China), Animal Tissue Total RNA Extraction and Purification Kit (DP431, TIANGEN, Beijing, China), Maxima H Minus First Strand cDNA Synthesis Kit (K1652, Thermo Scientific, United States), PowerUp SYBR Green Master Mix (A25741, Thermo Scientific, United States), 0.45 μm PVDF membrane (E-BC-R266, Thermo Scientific, United States), 0.2 μm NC membrane (RM02801, Abclonal, Wuhan, China). Primary antibodies included p38 rabbit polyclonal antibody (A0027, Abclonal, Wuhan, China), ERK rabbit polyclonal antibody (AP1120, Abclonal, Wuhan, China), JNK rabbit polyclonal antibody (A18678, Abclonal, Wuhan, China), zonulin rabbit polyclonal antibody (A1571, Abclonal, Wuhan, China), β-Actin rabbit monoclonal antibody (highly sensitive) (AC026, Abclonal, Wuhan, China), MUC1 rabbit polyclonal antibody (A21726, Abclonal, Wuhan, China), MUC2 rabbit polyclonal antibody (A14659, Abclonal, Wuhan, China), ZO-1 rabbit polyclonal antibody (A0659, Abclonal, Wuhan, China), occludin rabbit polyclonal antibody (A12621, Abclonal, Wuhan, China), claudin 1 rabbit polyclonal antibody (A21770, Abclonal, Wuhan, China), and HRP-conjugated sheep anti-rabbit IgG (H + L) (A0208, Abclonal, Wuhan, China). Other reagents were listed below: PBS buffer (BL601A, LABGIC, Beijing, China), 5× SDS-PAGE protein loading buffer (BL502B, LABGIC, Beijing, China), penicillin-streptomycin mixture for cell culture (P1400, Solarbio, Beijing, China), sodium chloride (10019318, Sinopharm Chemical Reagent Co., Ltd, Shanghai, China), SDS (EZ6470EE3A, Jinpan, Shanghai, China), glycine (EZ65A7898, Jinpan, Shanghai, China), skim milk powder (EZ63FCCF1C, Jinpan, Shanghai, China), Tris (EA63B43DD9, Jinpan, Shanghai, China), Annexin V-FITC/PI fluorescent double-stained apoptosis assay kit (WH01112305XP24, Pricella, Wuhan, China), Lipo3000 transfection reagent (L3000001, Thermo Scientific, United States), P3000 enhancer (L3000001, Thermo Scientific, United States), Opti-MEM reduced serum medium (31985070, Gibco, USA), and BCA protein concentration assay kit (PC0020, Solarbio, Beijing, China).

### Cell treatment

After constructing the Zonulin up-regulation cell line and the Zonulin knockdown cell line, the Zonulin up-regulation cell line and the Zonulin knockdown cell line were set up as follows: Negative control group (control group, 200 µL PBS buffer was added for 4 h), positive control group (model group, 200 uL 5 × 10^5^ CFU/mL *E.coli* O78 was added for 4 h), low-dose group (LLAB group, 200 uL 5 × 10^5^ CFU/mL *E.coli* O78 and 200 uL 5 × 10^5^ CFU/mL *Lactobacillus yoelii* Lac-2 were added for 4 h), High-dose lactobacillus group (HLAB group, 200 uL 5 × 10^5^ CFU/mL *E.coli* O78 and 200 uL 5 × 10^7^ CFU/mL *Lactobacillus yoelii* Lac-2 for 4 h). MAPK activator (MAPK activator (Aurintricarboxylic Acid), dissolved in DMSO, diluted to 50 µΜ, reagent added at 10 µM per well) and MAPK blocker (MAPK blocker (SC68376), dissolved in DMSO, diluted to 50 µM, reagent added at 40 µM per well) for 48 h.

### Construction of Zonulin up-regulation and Zonulin knockdown cell lines and addition of MAPK activators and MAPK blockers

The mRNA and protein expression levels of p38, ERK, JNK and Zonulin were detected by real-time quantitative reverse transcription polymerase chain reaction (RT-qPCR) and Western blotting. The Zonulin up-regulation plasmid vector and Zonulin knockdown siRNA sequences synthesized by Qingke Biotechnology Co., Ltd. were transfected according to the corresponding transfection system (Table 1), and the total RNA and total protein were extracted by adding MAPK activator and MAPK blocker. Concentrations were determined using NanoDrop One and BCA, and transcribed into cDNA and protein loading buffer, respectively. RT-qPCR and Western blotting were performed, and the primer sequences were shown in Table 2.

**Table 1:** Cell transfection system.

	Name of the reagent	Zonulin up-regulation transfection system	siRNA transfection system
		Volume/well	
Dilute the transfection reagent	Opti-MEM	125 µL	125 µL
	Lipo 3000	7.5 µL	7.5 µL
Dilute the DNA/siRNA	Opti-MEM	125 µL	125 µL
	DNA/siRNA-1	5 µg	75 pmol
	P3000	10 µL	

**Table 2:** Real-time PCR primers.

Primer name	Primer sequences	Product length
P38-F	GATTTCGGACTGGCTCGACA	278
P38-R	TGGCCACAACCTGTTTGAGA	
ERK-F	ACATTCCTAAGGCTCAAGGGC	220
ERK-R	CAGAGCCAGGACCATCCATAG	
JNK-F	GTGGGGTGCATTATGGGAGAA	291
JNK-R	AGATGCGTCGATTACCAGCA	
MUC1-F	TTGCGCTGGCCATCATCTAT	237
MUC1-R	AAGTGGCTGCCAGGTTTGTA	
MUC2-F	AAGCAGACCTGCCTGAAGAC	108
MUC2-R	CAGGTTCACCGTCTGCTCAT	
ZO-1-F	GACCATCGTCTGCGCTATGA	565
ZO-1-R	CTCTGGTGAGCACGGATTGT	
Occludin-F	GTTTGGTTCGCTGGGAGAAGA	200
Occludin-R	CATTGGTCGAACGTGCATCTC	
Claudins-F	TTTACTCCTATGCCGGCGAC	173
Claudins-R	GAGGATGCCAACCACCATCA	
Zonulin-F	CCAAGTACCAGGACGACACC	131
Zonulin-R	ACCATACTCAGCCACAGCAC	
GAPDH-F	ACAGTCAAGGCAGAGAACGG	240
GAPDH-R	CTCGTGGTTCACACCCATCA	

### Intestinal epithelial cell barrier function detection

Transmembrane resistance (TEER), FITC-D4 flux, and bacterial transport were used to evaluate the permeability of the epithelial cell barrier for Zonulin up-regulation cell lines and Zonulin knockdown cell lines. Transmembrane resistance is measured with a resistive system (Millipore, Massachusetts, United States). Measure the fluorescence values of the lower chamber culture 2 h after the addition of FITC-D4 to the upper chamber of each group. After 4 h, the lower chamber culture was collected to calculate colony-forming units (CFU) to quantify bacterial transport.

### Detection of mRNA expression levels of mucin and TJ protein in intestinal epithelial cells

The mRNA expression levels of mucin (MUC1, MUC2) and TJ proteins (ZO-1, Occludin, Claudin 1, Zonulin) were detected by real-time quantitative reverse transcription polymerase chain reaction (RT-qPCR) in Zonulin up-regulation cell lines and Zonulin knockdown cell lines. Total RNA from each treatment group was extracted using the RNA-easy Isolation Reagent Kit and its purity, integrity, and concentration were measured with the NanoDrop One. Complementary DNA was synthesized from 1 mg of total RNA following the kit instructions using the first-strand cDNA synthesis kit. The primers were synthesized by Tsingke Biotechnology (Tsingke, Beijing, China), and the primer sequences are shown in Table 2.

### Detection of protein expression levels of mucin and TJ protein in intestinal epithelial cells

Western blotting was used to detect the protein expression levels of mucin (MUC1, MUC2) and TJ proteins (ZO-1, Occludin, Claudin 1, Zonulin) in Zonulin up-regulation cell lines and Zonulin knockdown cell lines. Total protein was extracted using RIPA lysate RIPA lysis buffer, protein concentration was determined by BCA, and protein loading buffer was prepared by adding 5x SDS-PAGE protein loading buffer. The total protein of each group was separated using 10% SDS-PAGE, the protein was transferred to the NC membrane by wet transfer, and sealed with 5% skim milk on a shaker for 1 h. After 1 h of primary antibody incubation, wash the membrane 5 times with 1x TBST; Incubate with secondary antibody for 1 h and wash the membrane with 1x TBST for 5 more times. The gray value of the band was determined using chemiluminescence with β-actin as the internal reference. Grayscale values of protein bands were quantified and normalized using ImageJ 1.54f (https://imagej.nih.gov/ij/).

### Detection of apoptosis in intestinal epithelial cells

Flow cytometry was used to detect the apoptosis of Zonulin up-regulation cell lines and Zonulin knockdown cell lines in each treatment group. Collect 1 ~ 5 × 105 cells per group, collect the cell pellet by centrifugation, wash twice with PBS buffer, and add 100 µL of diluted 1xAnnexin V Binding Buffer to resuspend the cells. According to the instructions of the Annexin V-FITC/PI kit (Pricella, WH01112305XP24, Wuhan, China), Annexin V-FITC staining solution (2.5 µL) and Annexin V-PI staining solution (2.5 µL) were added to the cell suspension, mixed well, and incubated at room temperature for 10 ~ 15 min in the dark. Tests are performed on the machine immediately after staining.

### Statistical analysis of data

Each group of experiments was repeated more than 3 times, and the data were expressed as mean ± standard deviation. A one-way ANOVA was performed on all data using GraphPadPrism 9.0, and then the mean values of each group were compared using the Bonferroni test. *p* < 0.05, the difference was considered statistically significant.

## Results

### Construction of Zonulin up-regulation and Zonulin knockdown cell lines and addition of MAPK activators and MAPK blockers

**Fig. 1:**
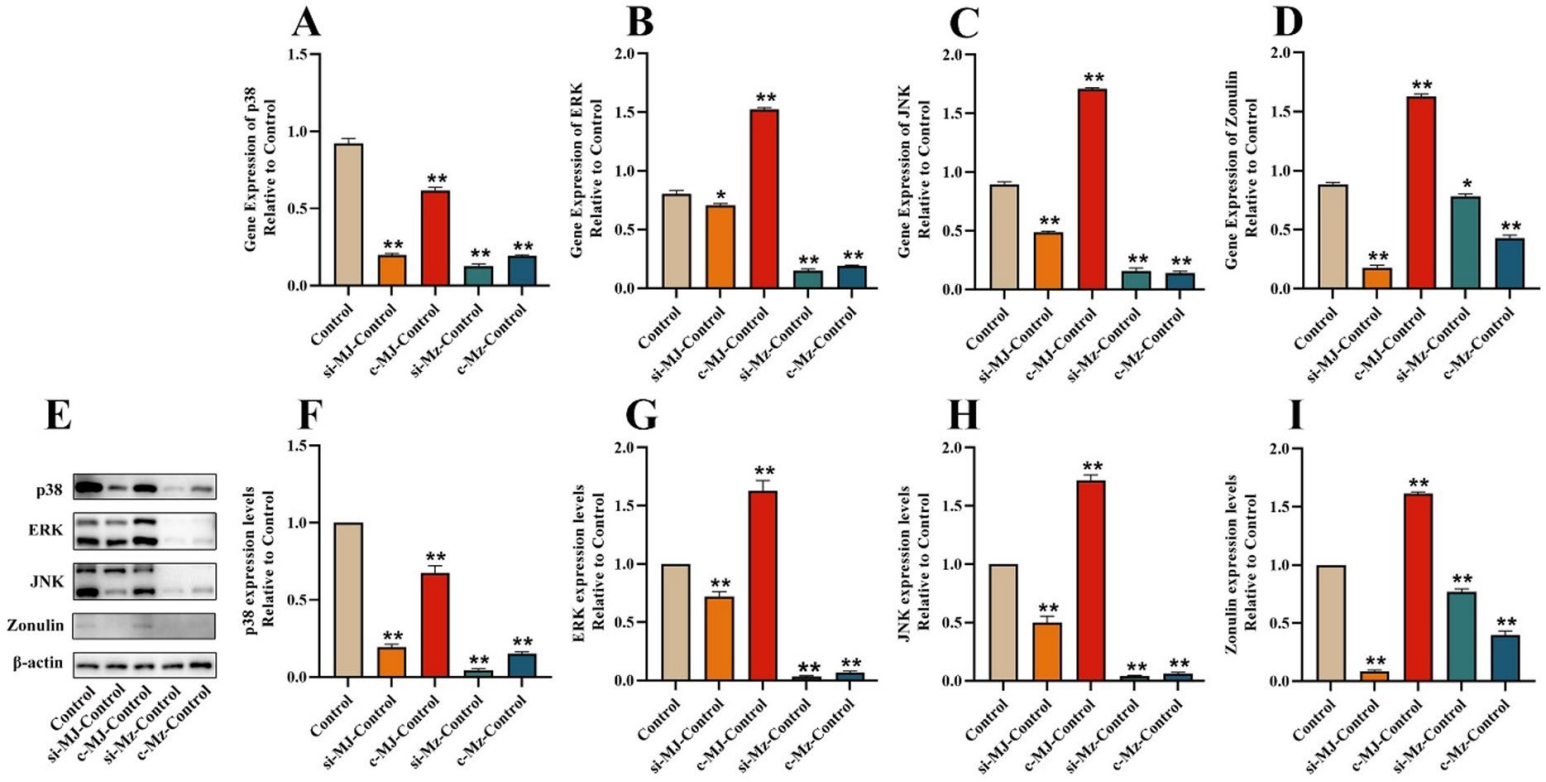
Construction of Zonulin up-regulation and Zonulin knockdown cell lines, as well as mRNA and protein expression levels with the addition of MAPK activator and MAPK blocker. (**A**) Relative expression of p38 mRNA; (**B**) Relative expression of ERK mRNA; (**C**) Relative expression of JNK mRNA; (**D**) Relative expression of Zonulin mRNA; (**E**) WB gray value detection results; (**F**) Relative β-actin protein expression level of p38; (**G**) Relative β-actin protein expression level of ERK; (**H**) Relative β-actin protein expression level of JNK; (**I**) Relative β-actin protein expression levels of Zonulin. *p < 0.05, **p < 0.01.

Figure 1 shows the mRNA and protein expression levels of intestinal epithelial cells p38, ERK, JNK, and Zonulin from intestinal epithelial cells. Compared with the control group, the mRNA and protein expression levels of p38, JNK and Zonulin in the si-MJ-Control group were significantly lower than those in the Control group (*p* < 0.01). The mRNA expression level of ERK in the si-MJ-Control group was significantly lower than that in the Control group (*p* < 0.05), and the protein expression level of ERK in the si-MJ-Control group was significantly lower than that in the Control group (*p* < 0.01). Compared with the control group, the mRNA and protein expression levels of p38, ERK, JNK and Zonulin in the c-MJ-Control group were significantly higher than those in the Control group (*p* < 0.01). Compared with the control group, the mRNA and protein expression levels of p38, ERK, JNK and Zonulin in the si-Mz-Control group were significantly lower than those in the Control group (*p* < 0.01). Compared with the Control group, the mRNA and protein expression levels of p38, ERK, JNK and Zonulin in the c-Mz-Control group were significantly lower than those in the Control group (*p* < 0.01). From this, we can see that the Zonulin up-regulation and Zonulin knockdown cell lines were successfully constructed, as well as the MAPK activator and MAPK blocker were also successfully added.

### Intestinal epithelial barrier function assay results of Zonulin overexpressing cell line with MAPK activator

**Fig. 2:**
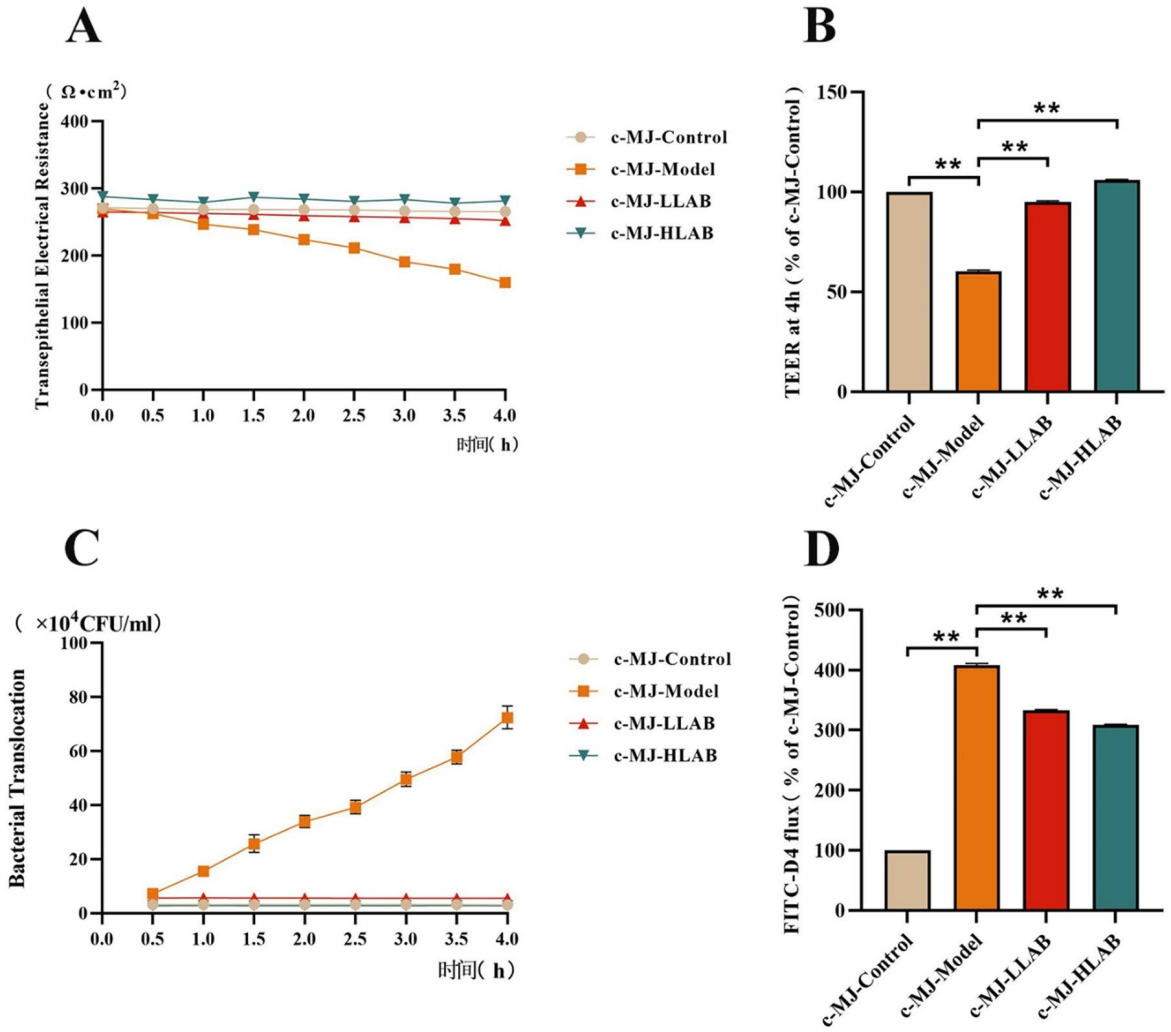
The results of epithelial barrier function in each group of Zonulin overexpressing cell lines after the addition of MAPK activator. (**A**) Changes in the level of transmembrane resistance within 4 h in each treatment group; (**B**) Percentage of 4-h transmembrane resistance in each treatment group relative to the level of the c-MJ-Control group; (**C**) Changes in bacterial translocation levels in each treatment group within 4 h; (**D**) Percentage of FITC-D4 permeability relative to the level of the c-MJ-Control group in each treatment group. *p < 0.05, **p < 0.01.

Compared with the c-MJ-Control group, the transmembrane resistance (TEER) in the c-MJ-Model group showed a downward trend within 4 h, and decreased significantly within 1–4 h (*p* < 0.01). The transmembrane resistance in the c-MJ-LLAB and c-MJ-HLAB groups was consistently greater than 200 Ω cm^2^ and remained at a normal level (Fig. 2A). Compared with the c-MJ-Control group, the percentage of transmembrane resistance in the c-MJ-Model group at the 4th hour in the c-MJ-Model group was significantly lower than that in the c-MJ-Control group (*p* < 0.01). Compared with the c-MJ-Model group, the c-MJ-LLAB group and the c-MJ-HLAB group had a very significant increase (*p* < 0.01) and showed a dependence on the dose of *Lactobacillus yoelii* Lac-2 (Fig. 2B). Compared with the c-MJ-Control group, the amount of bacterial translocation in the c-MJ-Model group increased significantly within 4 h (*p* < 0.01). Compared with the c-MJ-Model group, the bacterial translocation in the c-MJ-LLAB group and the c-MJ-HLAB group was significantly reduced within 1–4 h (*p* < 0.01), and also showed a dependence on the dose of *Lactobacillus yoelii* Lac-2 (Fig. 2C). After 4 h of bacterial solution, the FD4 permeability of the c-MJ-Model group was significantly higher than that of the c-MJ-Control group (*p* < 0.01). Compared with the c-MJ-Model group, the FD4 permeability of the c-MJ-LLAB group and the c-MJ-HLAB group was significantly reduced (*p* < 0.01) and showed a dependence on the dose of *Lactobacillus yoelii* Lac-2 (Fig. 2D).

### Intestinal epithelial barrier function of Zonulin overexpressing cell line with MAPK blocker

**Fig. 3:**
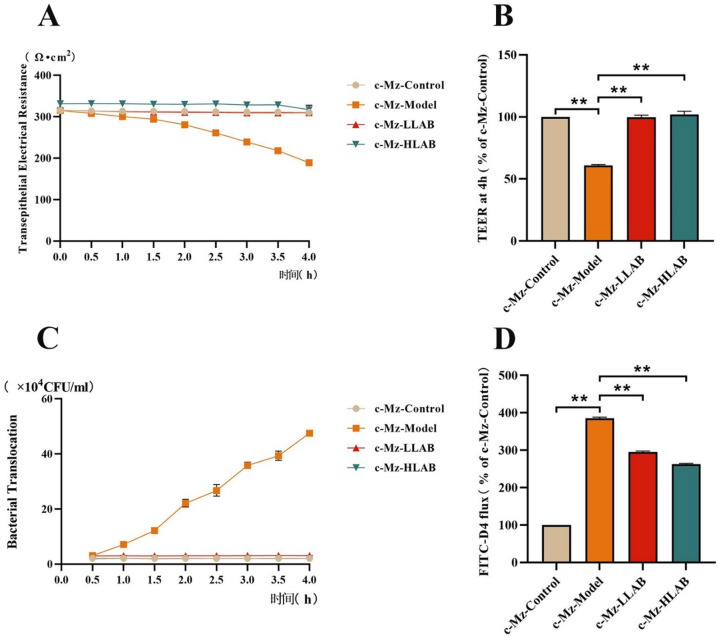
The epithelial barrier function of Zonulin overexpressing cell lines after the addition of MAPK blocker was detected. (**A**) Changes in the level of transmembrane resistance within 4 h in each treatment group; (**B**) Percentage of 4-h transmembrane resistance in each treatment group relative to the level of the c-Mz-Control group; (**C**) Changes in bacterial translocation levels in each treatment group within 4 h; (**D**) Percentage of FITC-D4 permeability relative to the level of the c-Mz-Control group in each treatment group. *p < 0.05, **p < 0.01.

Compared with the c-Mz-Control group, the transmembrane resistance (TEER) of the c-Mz-Model group showed a downward trend within 4 h, and decreased significantly within 1–4 h (*p* < 0.01). The transmembrane resistance in the c-Mz-LLAB and c-Mz-HLAB groups was consistently greater than 200 Ω cm^2^ and remained at normal levels (Fig. 3A). Compared with the c-Mz-Control group, the percentage of transmembrane resistance in the c-Mz-Model group at the 4th hour in the c-Mz-Model group was significantly lower (*p* < 0.01). Compared with the c-Mz-Model group, the c-Mz-LLAB group and the c-Mz-HLAB group had a very significant increase (*p* < 0.01) and showed a dependence on the dose of *Lactobacillus yoelii* Lac-2 (Fig. 3B). Compared with the c-Mz-Control group, the amount of bacterial translocation in the c-Mz-Model group increased significantly within 1–4 h (*p* < 0.01). Compared with the c-Mz-Model group, the amount of bacterial translocation in the c-Mz-LLAB group and the c-Mz-HLAB group was significantly reduced within 1–4 h (*p* < 0.01), and it also showed a dependence on the dose of *Lactobacillus yoelii* Lac-2 (Fig. 3C). After 4 h of addition of the bacterial solution, the FD4 permeability in the c-Mz-Model group was significantly higher than that in the c-Mz-Control group (*p* < 0.01). Compared with the c-Mz-Model group, the FD4 permeability was significantly reduced in the c-Mz-LLAB and c-Mz-HLAB groups (*p* < 0.01) and showed a dependence on the dose of *Lactobacillus yoelii* Lac-2 (Fig. 3D).

### Intestinal epithelial barrier function assay results of Zonulin knockdown cell line with MAPK activator

**Fig. 4:**
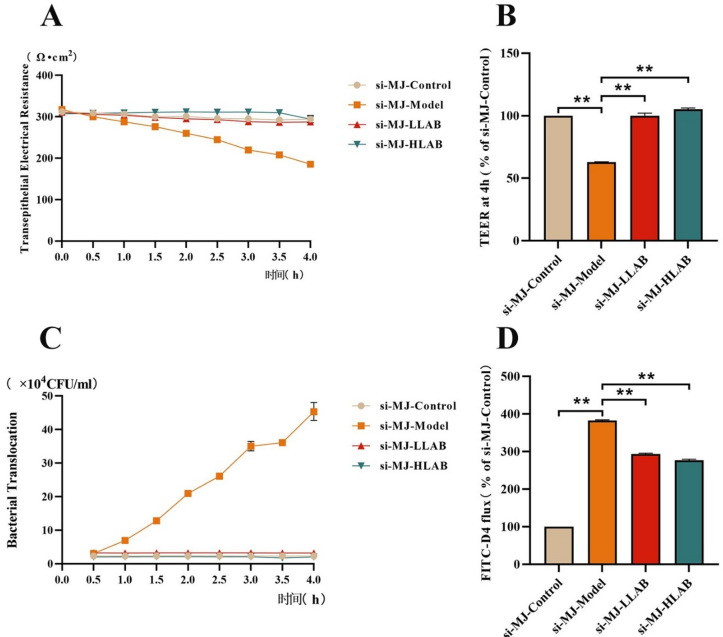
Epithelial barrier function of Zonulin knockdown cell line after the addition of MAPK activator was detected. (**A**) Changes in the level of transmembrane resistance within 4 h in each treatment group; (**B**) Percentage of 4-h transmembrane resistance in each treatment group relative to the level of the si-MJ-Control group; (**C**) Changes in bacterial translocation levels in each treatment group within 4 h; (**D**) Percentage of FITC-D4 permeability relative to the level of the si-MJ-Control group in each treatment group. *p < 0.05, **p < 0.01.

Compared with the si-MJ-Control group, the transmembrane resistance (TEER) of the si-MJ-Model group showed a downward trend within 4 h, and decreased significantly within 0.5–4 h (*p* < 0.01). The transmembrane resistance in the si-MJ-LLAB group and the si-MJ-HLAB group was always greater than 200 Ω cm^2^ and remained at a normal level (Fig. 4A). Compared with the si-MJ-Control group, the percentage of transmembrane resistance in the si-MJ-Model group at the 4th hour in the si-MJ-Model group was significantly lower than that in the si-MJ-Control group (*p* < 0.01). Compared with the si-MJ-Model group, the SI-MJ-LLAB group and the SI-MJ-HLAB group had a very significant increase (*p* < 0.01) and showed a dependence on the dose of *Lactobacillus yoelii* Lac-2 (Fig. 4B). Compared with the si-MJ-Control group, the amount of bacterial translocation in the si-MJ-Model group increased significantly within 1–4 h (*p* < 0.01). Compared with the si-MJ-Model group, the bacterial translocation in the si-MJ-LLAB and si-MJ-HLAB groups was significantly reduced within 1–4 h (*p* < 0.01), and also showed a dependence on the dose of *Lactobacillus yoelii* Lac-2 (Fig. 4C). After 4 h of bacterial solution, the FD4 permeability of the si-MJ-Model group was significantly higher than that of the si-MJ-Control group (*p* < 0.01). Compared with the si-MJ-Model group, the FD4 permeability of the si-MJ-LLAB group and the si-MJ-HLAB group was significantly reduced (*p* < 0.01) and showed a dependence on the dose of *Lactobacillus yoelii* Lac-2 (Fig. 4D).

### Intestinal epithelial barrier function of Zonulin knockdown cell line with MAPK blocker

**Fig. 5:**
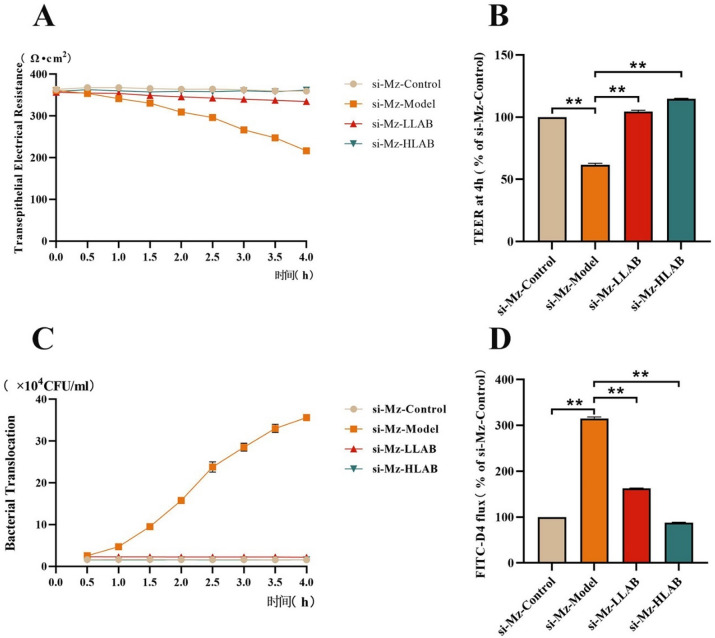
Epithelial barrier function of Zonulin knockdown cell line after MAPK blockade was added to each group. (**A**) Changes in the level of transmembrane resistance within 4 h in each treatment group; (**B**) Percentage of 4-h transmembrane resistance in each treatment group relative to the level of the si-Mz-Control group; (**C**) Changes in bacterial translocation levels in each treatment group within 4 h; (**D**) Percentage of FITC-D4 permeability relative to the level of the si-Mz-Control group in each treatment group. *p < 0.05, **p < 0.01.

Compared with the si-Mz-Control group, the transmembrane resistance (TEER) of the si-Mz-Model group showed a decreasing trend within 4 h, and decreased significantly within 0.5–4 h (*p* < 0.01). The transmembrane resistance in the si-Mz-LLAB and si-Mz-HLAB groups was consistently greater than 200 Ω cm^2^ and remained at normal levels (Fig. 5A). Compared with the si-Mz-Control group, the percentage of transmembrane resistance at the 4th hour in the si-Mz-Model group was significantly lower than that in the si-Mz-Control group (*p* < 0.01). Compared with the si-Mz-Model group, the si-Mz-LLAB group and the si-Mz-HLAB group had a very significant increase (*p* < 0.01) and showed a dependence on the dose of *Lactobacillus yoelii* Lac-2 (Fig. 5B). Compared with the si-Mz-Control group, the amount of bacterial translocation in the si-Mz-Model group increased significantly within 1–4 h (*p* < 0.01). Compared with the si-Mz-Model group, the amount of bacterial translocation in the si-Mz-LLAB group and the si-Mz-HLAB group was significantly reduced within 1–4 h (*p* < 0.01), and it also showed a dependence on the dose of *Lactobacillus yoelii* Lac-2 (Fig. 5C). After 4 h of bacterial solution, the FD4 permeability of the si-Mz-Model group was significantly higher than that of the si-Mz-Control group (*p* < 0.01). Compared with the si-Mz-Model group, the FD4 permeability of the si-Mz-LLAB and si-Mz-HLAB groups was significantly reduced (*p* < 0.01) and showed a dependence on the dose of *Lactobacillus yoelii* Lac-2 (Fig. 5D).

### Intestinal epithelial barrier function in the HLAB group of Zonulin up-regulation and Zonulin knockdown cell lines with MAPK activator and MAPK blocker

**Fig. 6:**
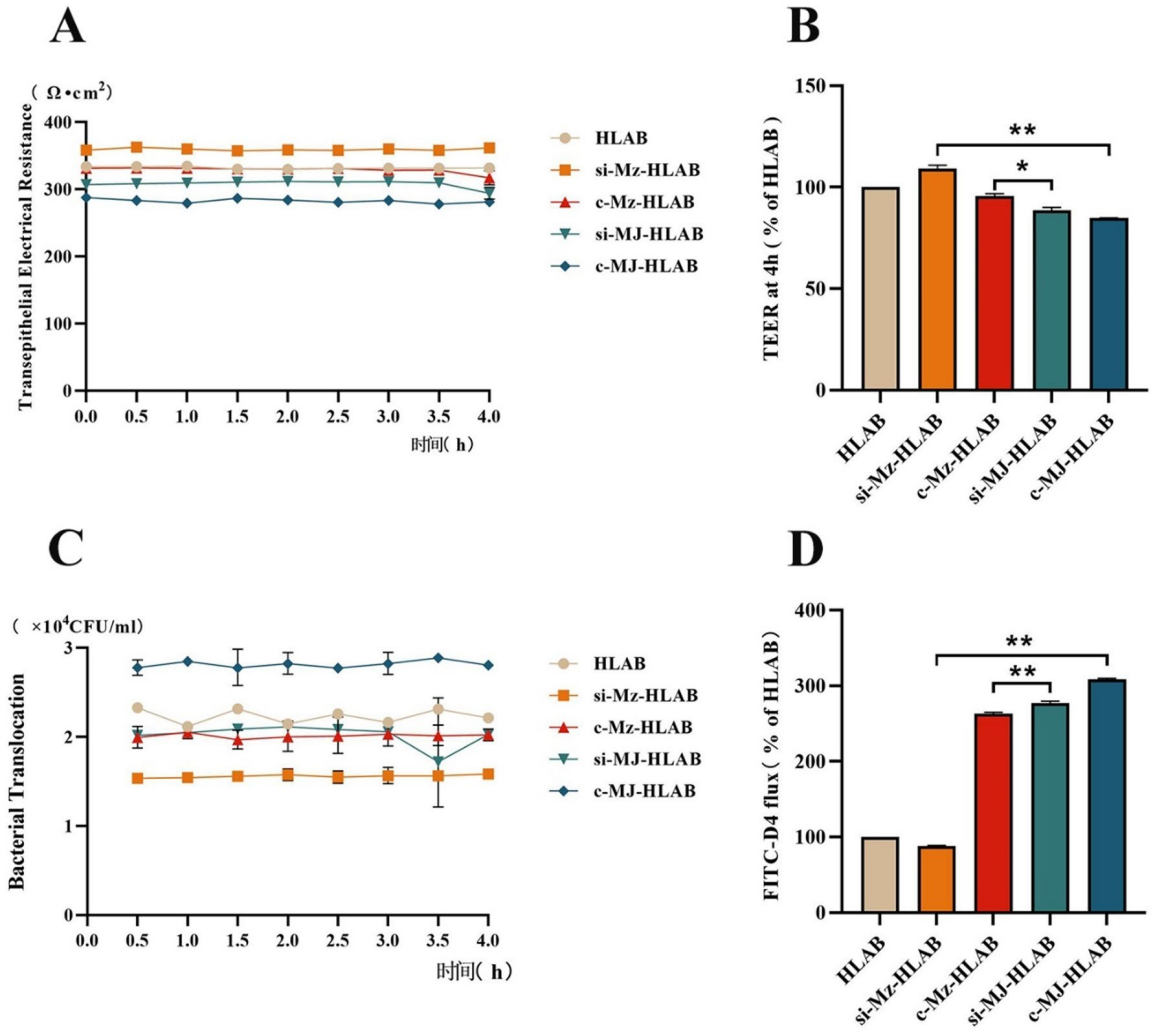
Epithelial barrier function of Zonulin up-regulation and Zonulin knockdown cell lines after the addition of MAPK activator and MAPK blocker were detected. (**A**) Changes in the level of transmembrane resistance within 4 h in each treatment group; (**B**) Percentage of 4-h transmembrane resistance in each treatment group relative to the level of the HLAB group; (**C**) Changes in bacterial translocation levels in each treatment group within 4 h; (**D**) Percentage of FITC-D4 permeability relative to the level of the HLAB group in each treatment group. *p < 0.05, **p < 0.01.

The transmembrane resistance (TEER) of the si-Mz-HLAB group was higher than that of the c-MJ-HLAB group within 4 h. The transmembrane resistance (TEER) within 4 h in the c-Mz-HLAB group was also higher than that in the si-MJ-HLAB group. The transmembrane resistance of all experimental groups with high doses of *Lactobacillus yoelii* Lac-2 was always greater than 200 Ω cm^2^ and remained at a normal level (Fig. 6A). Compared with the c-MJ-HLAB group, the percentage of transmembrane resistance in the 4th h of the si-Mz-HLAB group increased significantly compared with that of the c-MJ-HLAB group (*p* < 0.01). The percentage of transmembrane resistance at the 4th h in the c-Mz-HLAB group was significantly higher than that in the SI-MJ-HLAB group (*p* < 0.01) (Fig. 6B). The amount of bacterial translocation within 4 h in the c-MJ-HLAB group was higher than that in the si-Mz-HLAB group, and there was no significant difference in bacterial translocation within 4 h in the c-MZ-HLAB group and the si-MJ-HLAB group. However, at 3.5 h, the c-Mz-HLAB group was higher than the si-MJ-HLAB group (Fig. 6C). After 4 h of bacterial solution, the FD4 permeability of the si-Mz-HLAB group was significantly higher than that of the c-MJ-HLAB group (*p* < 0.01), and the FD4 permeability of the c-Mz-HLAB group was significantly higher than that of the si-MJ-HLAB group (*p* < 0.01) (Fig. 6D).

### The mRNA expression levels of mucin and TJ protein in intestinal epithelial cells with MAPK activator in Zonulin up-regulation cell line

**Fig. 7:**
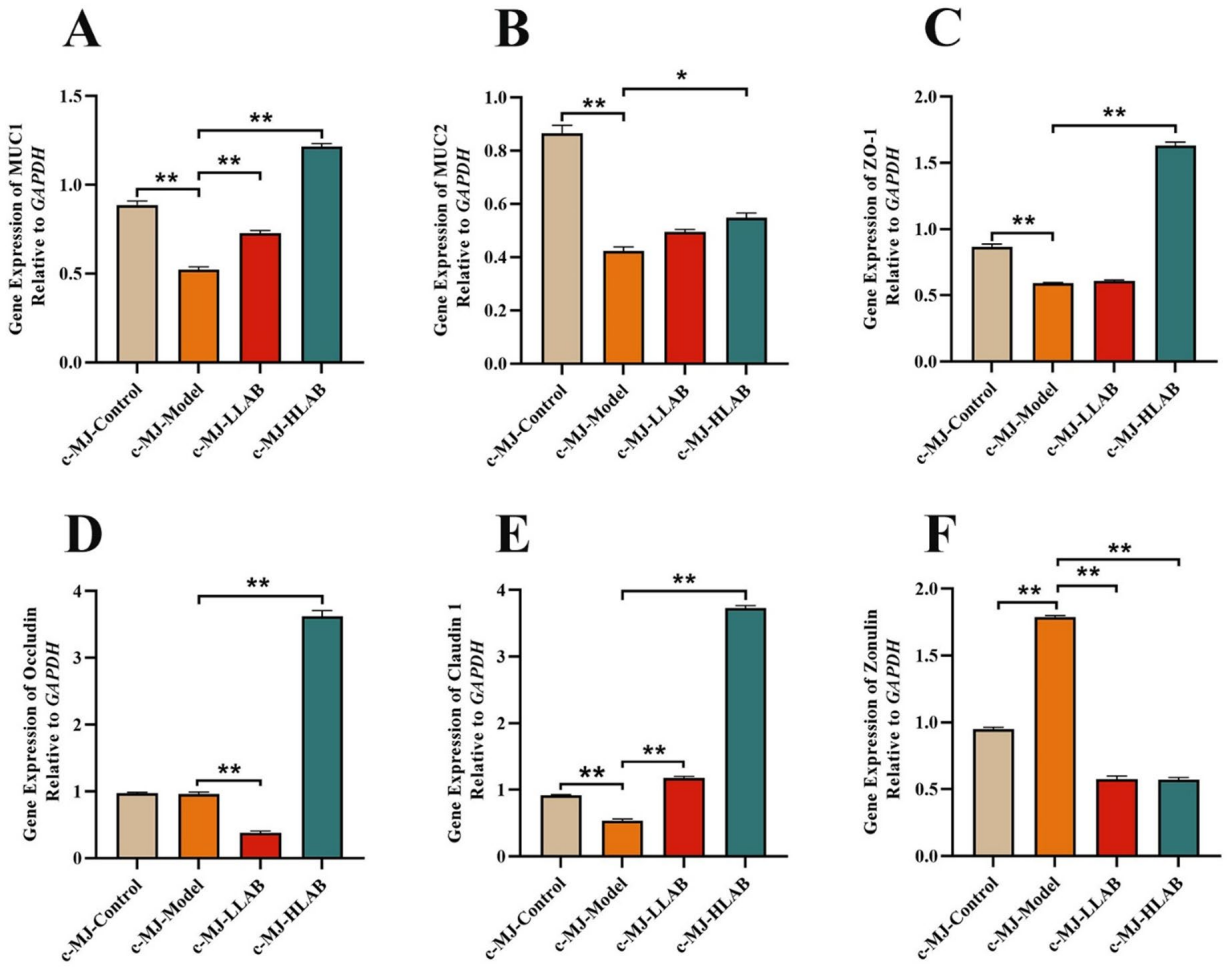
The mRNA expression levels of mucin and TJ proteins in each treatment group after the addition of MAPK activator to Zonulin overexpressing cell lines. (**A**) Relative expression of MUC1 mRNA; (**B**) Relative expression of MUC2 mRNA; (**C**) Relative expression of ZO-1 mRNA; (**D**) Relative mRNA expression of Occludin; (**E**) Relative expression of Claudin 1 mRNA; (**F**) Relative expression of Zonulin mRNA. *p < 0.05, **p < 0.01.

Figure 7 shows the RT-qPCR detection of mucin and TJ proteins in each group of intestinal epithelial cells treated with MAPK activator in Zonulin-overexpressed intestinal epithelial cell lines. The results showed that compared with the c-MJ-Control group, the mRNA levels of MUC1, MUC2, ZO-1 and Claudin 1 in the c-MJ-Model group were significantly decreased (*p* < 0.01), and the mRNA levels of Zonulin were significantly increased (*p* < 0.01). Compared with the c-MJ-Model group, the mRNA expression levels of MUC1, Occludin, Claudin 1 and Zonulin were significantly restored after the addition of low dose of *Lactobacillus yoelii* Lac-2 (*p* < 0.01). After the addition of high doses of *Lactobacillus yoelii* Lac-2, MUC1, ZO-1, Occludin, Claudin 1 and Zonulin were significantly restored (*p* < 0.01), and MUC2 was significantly restored (*p* < 0.05).

### The mRNA expression levels of mucin and TJ protein in intestinal epithelial cells with MAPK blocker added to Zonulin up-regulation cell line were detected

**Fig. 8:**
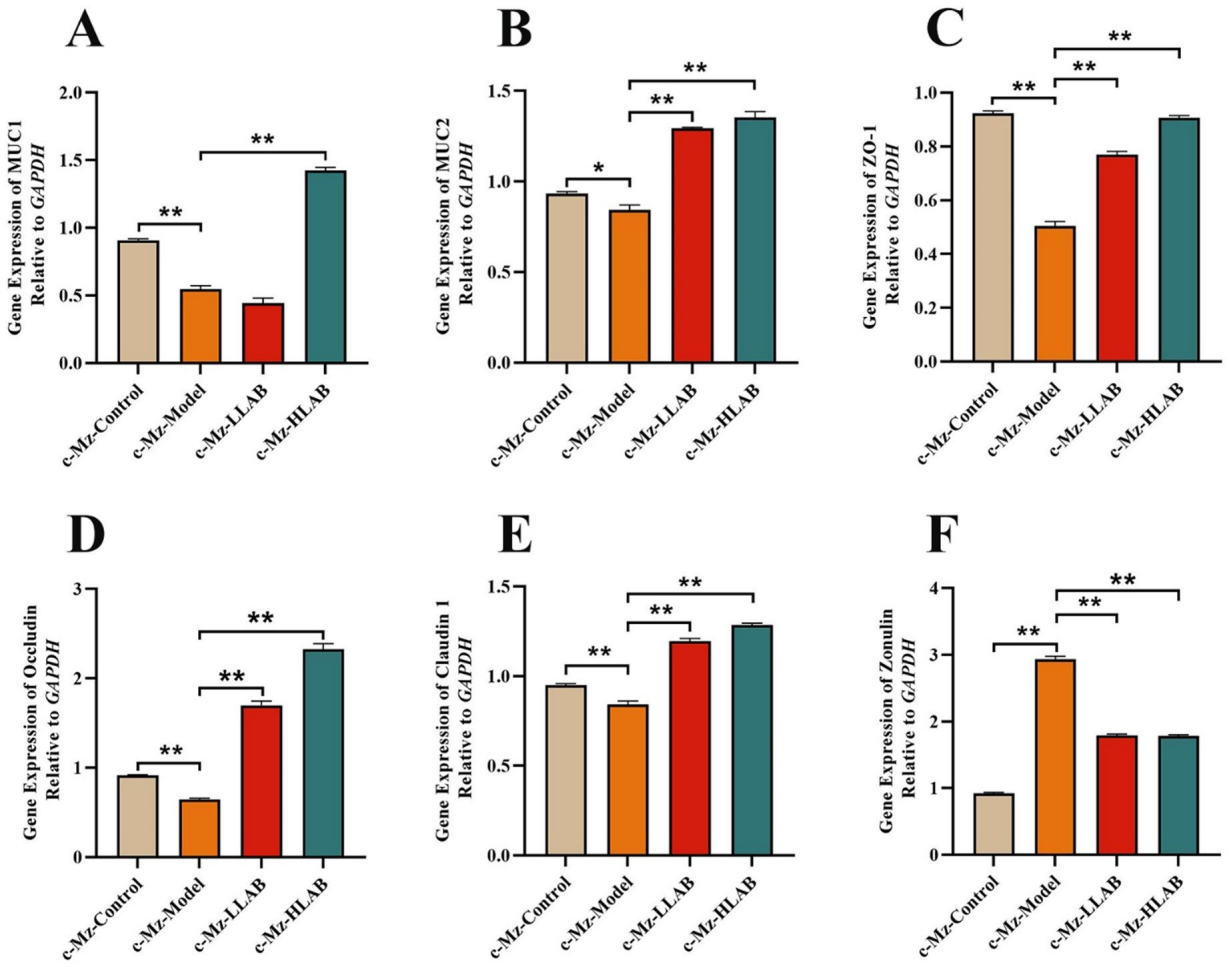
The mRNA expression levels of mucin and TJ proteins in each treatment group after the addition of MAPK blocker to the Zonulin up-regulation cell line. (**A**) Relative expression of MUC1 mRNA; (**B**) Relative expression of MUC2 mRNA; (**C**) Relative expression of ZO-1 mRNA; (**D**) Relative mRNA expression of Occludin; (**E**) Relative expression of Claudin 1 mRNA; (**F**) Relative expression of Zonulin mRNA. *p < 0.05, **p < 0.01.

Figure 8 shows RT-qPCR assays of mucin and TJ proteins in each group of intestinal epithelial cells treated with MAPK blocker in Zonulin-overexpressed intestinal epithelial cell lines. The results showed that compared with the c-Mz-Control group, the mRNA levels of MUC1, ZO-1, Occludin, and Claudin 1 in the c-Mz-Model group were significantly decreased (*p* < 0.01), the mRNA expression levels of MUC2 were significantly decreased (*p* < 0.05), and the mRNA levels of Zonulin were significantly increased (*p* < 0.01). Compared with the c-Mz-Model group, the mRNA expression levels of MUC2, ZO-1, Occludin, Claudin 1 and Zonulin were significantly restored after the addition of low dose of *Lactobacillus yoelii* Lac-2 (*p* < 0.01). MUC1, MUC2, ZO-1, Occludin, Claudin 1, and Zonulin were significantly restored after the addition of high doses of *Lactobacillus yoelii* Lac-2 (*p* < 0.01).

### The mRNA expression levels of mucin and TJ protein in intestinal epithelial cells with MAPK activator were detected in Zonulin knockdown cell line

**Fig. 9:**
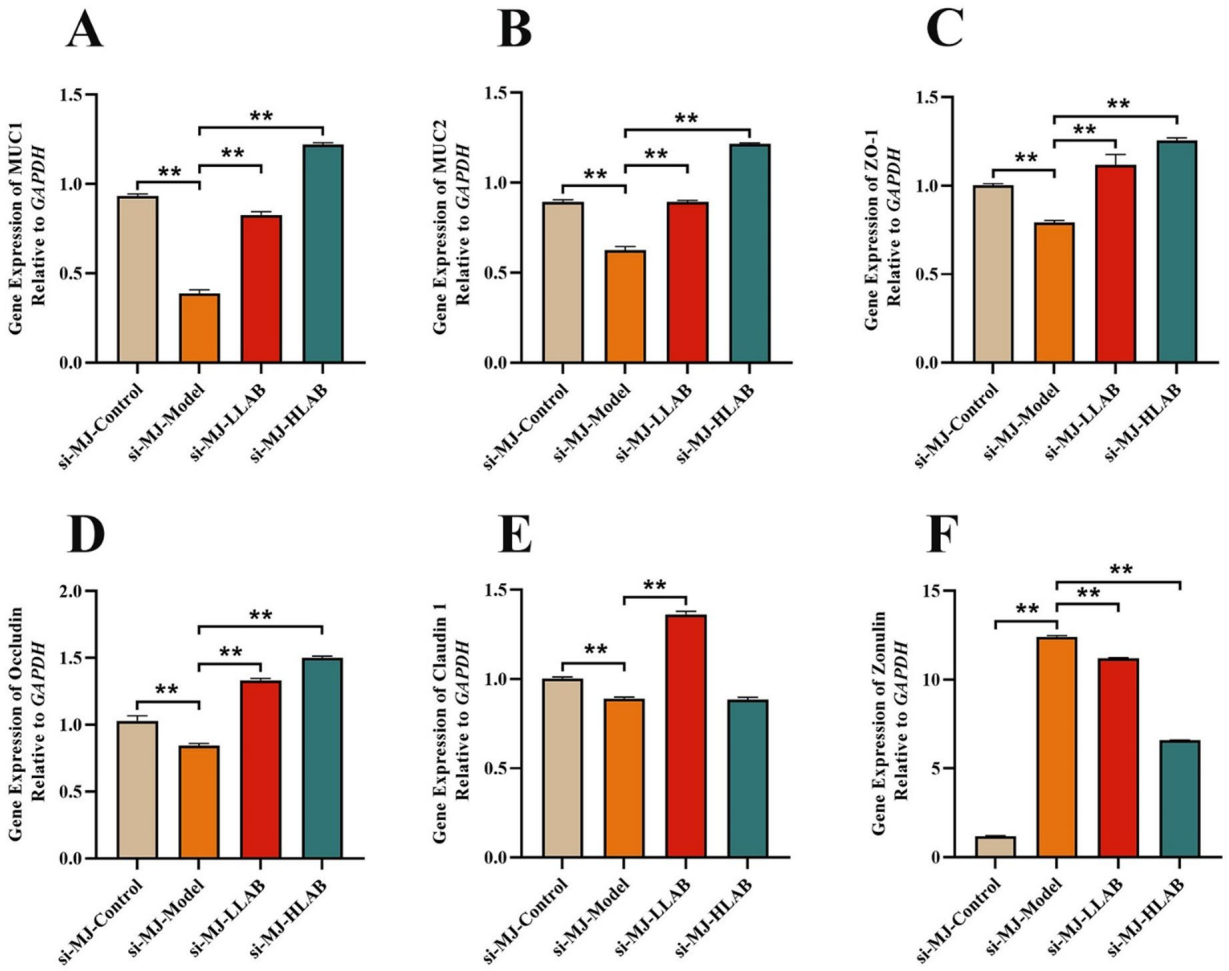
The mRNA expression levels of mucin and TJ protein in each treatment group were reduced by Zonulin knockdown cell line after MAPK activator was added. (**A**) Relative expression of MUC1 mRNA; (**B**) Relative expression of MUC2 mRNA; (**C**) Relative expression of ZO-1 mRNA; (**D**) Relative mRNA expression of Occludin; (**E**) Relative expression of Claudin 1 mRNA; (**F**) Relative expression of Zonulin mRNA. *p < 0.05, **p < 0.01.

Figure 9 shows RT-qPCR detection of mucin and TJ proteins in each group of intestinal epithelial cells treated with MAPK activator in a Zonulin-knocked intestinal epithelial cell line. The results showed that compared with the si-MJ-Control group, the mRNA levels of MUC1, MUC2, ZO-1, Occludin, and Claudin 1 in the si-MJ-Model group were significantly decreased (*p* < 0.01), and the mRNA levels of Zonulin were significantly increased (*p* < 0.01). Compared with the si-MJ-Model group, the mRNA expression levels of MUC1, MUC2, ZO-1, Occludin, Claudin 1 and Zonulin were significantly restored after the addition of low dose of *Lactobacillus yoelii* Lac-2 (*p* < 0.01). MUC1, MUC2, ZO-1, Occludin, and Zonulin were significantly restored after the addition of high doses of *Lactobacillus yoelii* Lac-2 (*p* < 0.01).

### The mRNA expression levels of mucin and TJ proteins in intestinal epithelial cells with MAPK blocker were detected by Zonulin knockdown cell line

**Fig. 10:**
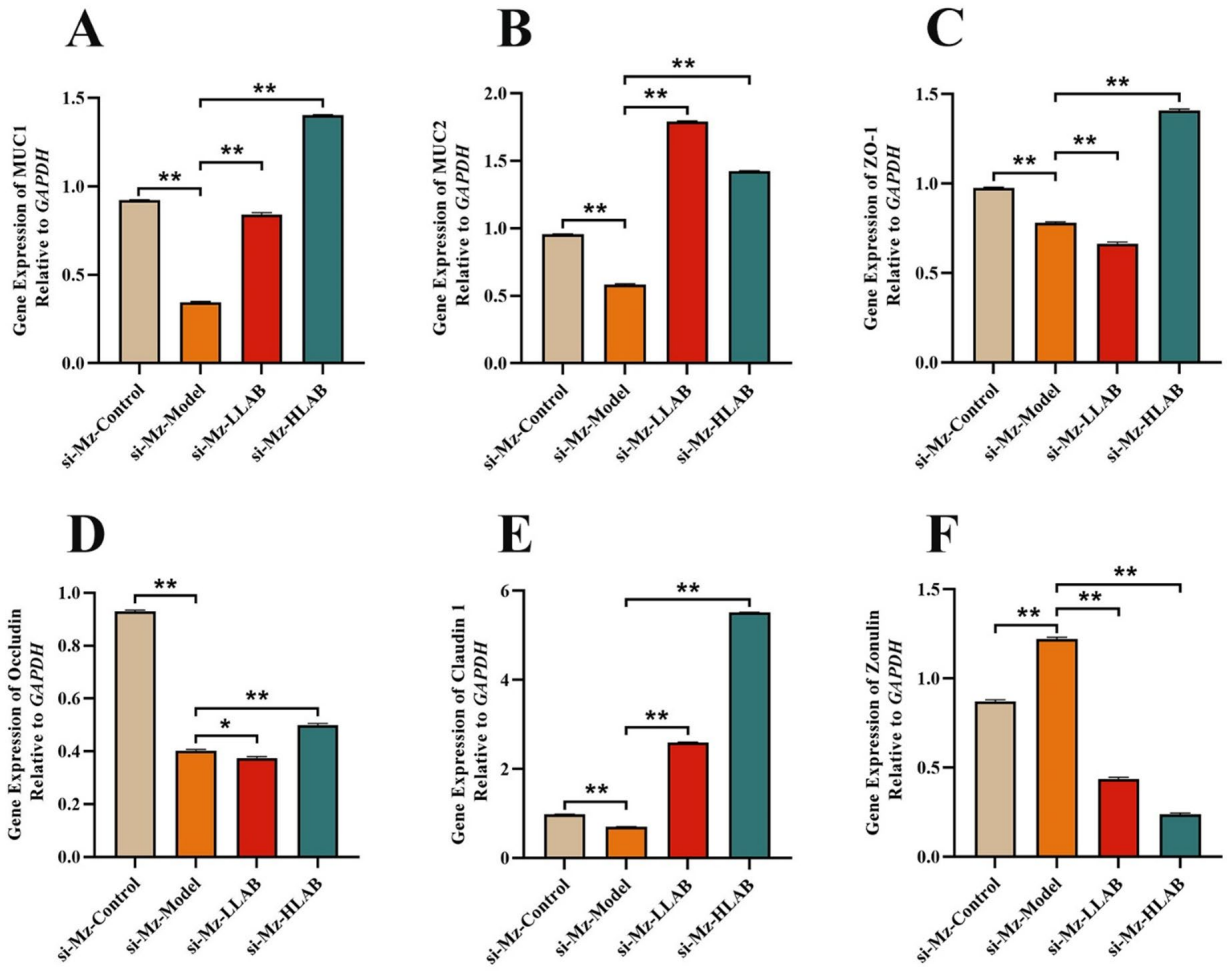
The mRNA expression levels of mucin and TJ proteins in each treatment group after Zonulin knockdown cell line was supplemented with MAPK blocker. (**A**) Relative expression of MUC1 mRNA; (**B**) Relative expression of MUC2 mRNA; (**C**) Relative expression of ZO-1 mRNA; (**D**) Relative mRNA expression of Occludin; (**E**) Relative expression of Claudin 1 mRNA; (**F**) Relative expression of Zonulin mRNA. *p < 0.05, **p < 0.01.

Figure 10 shows the RT-qPCR results of mucin and TJ proteins in each group of intestinal epithelial cells treated with MAPK blocker in a Zonulin-knocked intestinal epithelial cell line. The results showed that compared with the si-Mz-Control group, the mRNA levels of MUC1, MUC2, ZO-1, Occludin, and Claudin 1 in the si-Mz-Model group were significantly decreased (*p* < 0.01), and the mRNA levels of Zonulin were significantly increased (*p* < 0.01). Compared with the si-Mz-Model group, the mRNA expression levels of MUC1, MUC2, ZO-1, Claudin 1 and Zonulin were significantly restored after the addition of low dose of *Lactobacillus yoelii* Lac-2 (*p* < 0.01), and the mRNA expression levels of Occludin were significantly restored (*p* < 0.05). MUC1, MUC2, ZO-1, Occludin, Claudin 1, and Zonulin were significantly restored after the addition of high doses of *Lactobacillus yoelii* Lac-2 (*p* < 0.01).

### The mRNA expression levels of mucin and TJ protein in intestinal epithelial cells in the HLAB group of Zonulin up-regulation and Zonulin knockdown cell lines with MAPK activation and MAPK blocker were detected

**Fig. 11:**
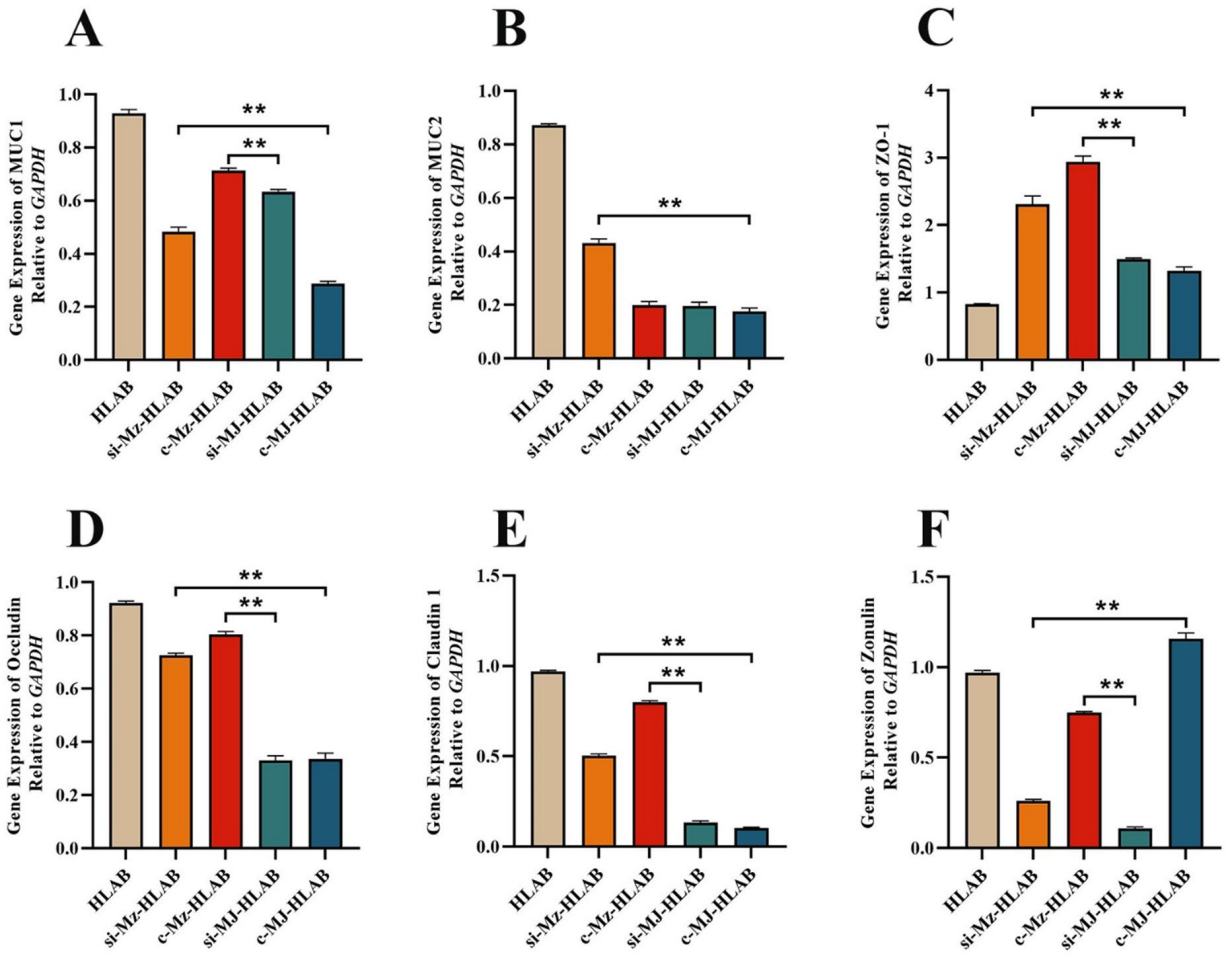
Zonulin up-regulation and Zonulin knockdown cell lines were treated with MAPK activator and MAPK blocker mRNA expression levels. (**A**) Relative expression of MUC1 mRNA; (**B**) Relative expression of MUC2 mRNA; (**C**) Relative expression of ZO-1 mRNA; (**D**) Relative mRNA expression of Occludin; (**E**) Relative expression of Claudin 1 mRNA; (**F**) Relative expression of Zonulin mRNA. *p < 0.05, **p < 0.01.

Figure 11 shows RT-qPCR detection of mucin and TJ proteins in intestinal epithelial cells treated with MAPK activator and MAPK blocker in Zonulin-overexpressing and Zonulin-knockdown intestinal epithelial cell lines. The results showed that compared with the c-MJ-HLAB group, the mRNA levels of MUC1, MUC2, ZO-1, Occludin, and Claudin 1 in the si-Mz-HLAB group were significantly increased (*p* < 0.01), and the mRNA levels of Zonulin were significantly decreased (*p* < 0.01). Compared with the si-MJ-HLAB group, the mRNA levels of MUC1, ZO-1, Occludin, Claudin 1 and Zonulin in the c-Mz-HLAB group were significantly increased (*p* < 0.01).

### Detection results of mucin and TJ protein in intestinal epithelial cells with MAPK activator in Zonulin up-regulation cell line

**Fig. 12:**
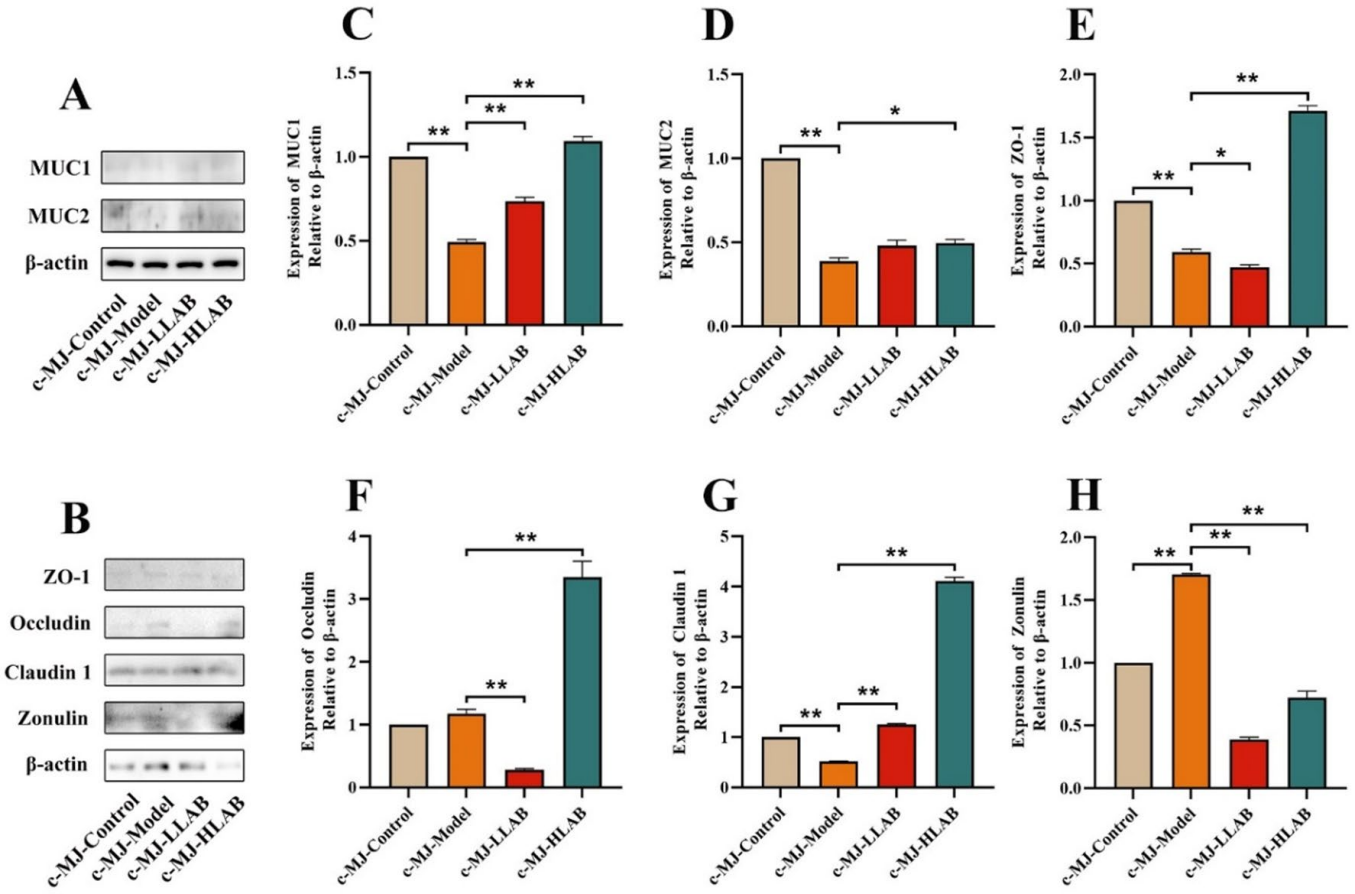
The expression levels of mucin and TJ protein in each treatment group after the addition of MAPK activator to Zonulin up-regulation cell line. (**A**) Gray value detection results of mucin WB; (**B**) Gray value of TJ protein WB; (**C**) Relative β-actin expression level of MUC1; (**D**) Relative β-actin expression level of MUC2; (**E**) Relative β-actin expression level of ZO-1; (**F**) Relative β-actin expression level of Occludin; (**G**) Claudin 1 relative β-actin expression level; (**H**) Relative β-actin expression levels of Zonulin. *p < 0.05, **p < 0.01.

Figure 12 shows Western bloting results of mucins and TJ proteins in intestinal epithelial cells treated with MAPK activator in Zonulin-overexpressing intestinal epithelial cell lines. The results showed that the protein expression levels of MUC1, MUC2, ZO-1 and Claudin 1 of c-MJ-Model were significantly decreased compared with those of c-MJ-Control (*p* < 0.01), and the protein expression levels of Zonulin were significantly increased (*p* < 0.01). The aberrant expression of these proteins was restored to a certain extent after the addition of *Lactobacillus yoelii* Lac-2. MUC1, Occludin, Claudin 1, and Zonulin were significantly restored with low-dose *Lactobacillus yoelii* Lac-2 (*p* < 0.01), and ZO-1 was significantly restored (*p* < 0.05). MUC1, ZO-1, Occludin, Claudin 1, and Zonulin were significantly restored (*p* < 0.01) and MUC2 (*p* < 0.05) with high doses of *Lactobacillus yoelii* Lac-2.

### Detection results of mucin and TJ protein levels of intestinal epithelial cells with MAPK blocker in Zonulin up-regulation cell line

**Fig. 13:**
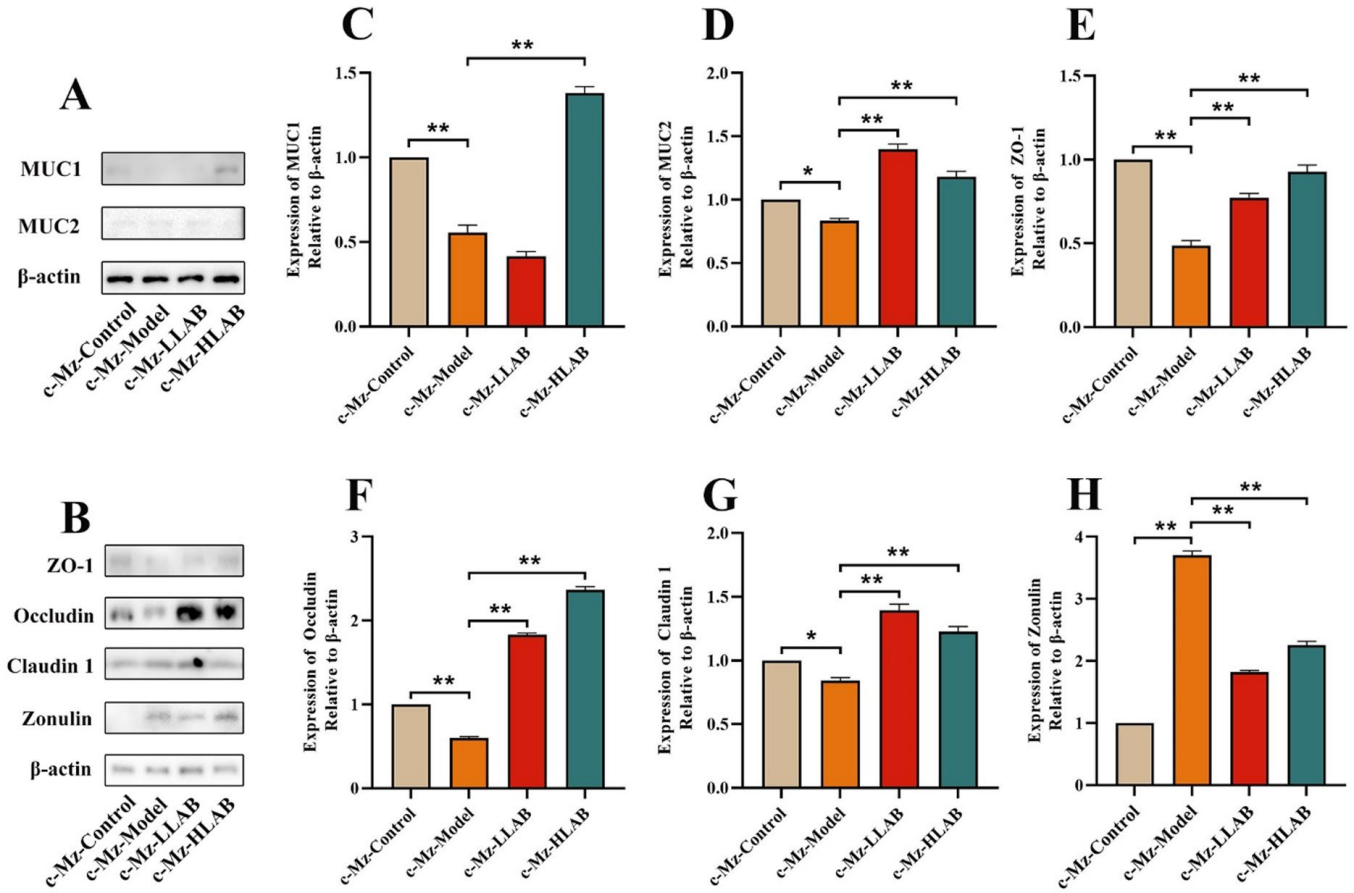
Expression levels of mucin and TJ protein in each treatment group after the addition of MAPK blocker to Zonulin up-regulation cell line. (**A**) Gray value detection results of mucin WB; (**B**) Gray value of TJ protein WB; (**C**) Relative β-actin expression level of MUC1; (**D**) Relative β-actin expression level of MUC2; (**E**) Relative β-actin expression level of ZO-1; (**F**) Relative β-actin expression level of Occludin; (**G**) Claudin 1 relative β-actin expression level; (**H**) Relative β-actin expression levels of Zonulin. *p < 0.05, **p < 0.01.

Figure 13 shows the Western bloting results of mucin and TJ proteins in intestinal epithelial cells in each group treated with MAPK blocker in the Zonulin-overexpressing intestinal epithelial cell line. The results showed that the protein expression levels of MUC1, ZO-1 and Occludin in c-Mz-Model were significantly decreased compared with c-Mz-Control (*p* < 0.01), MUC2 and Claudin 1 were significantly decreased (*p* < 0.05), and Zonulin was significantly increased (*p* < 0.01). The aberrant expression of these proteins was restored to a certain extent after the addition of *Lactobacillus yoelii* Lac-2. MUC2, ZO-1, Occludin, Claudin 1, and Zonulin were significantly restored with low-dose *Lactobacillus yoelii* Lac-2 (*p* < 0.01). MUC1, MUC2, ZO-1, Occludin, Claudin 1, and Zonulin were significantly restored with high doses of *Lactobacillus yoelii* Lac-2 (*p* < 0.01).

### Detection results of protein expression levels of mucin and TJ protein in intestinal epithelial cells with MAPK activator added to Zonulin knockdown cell line

**Fig. 14:**
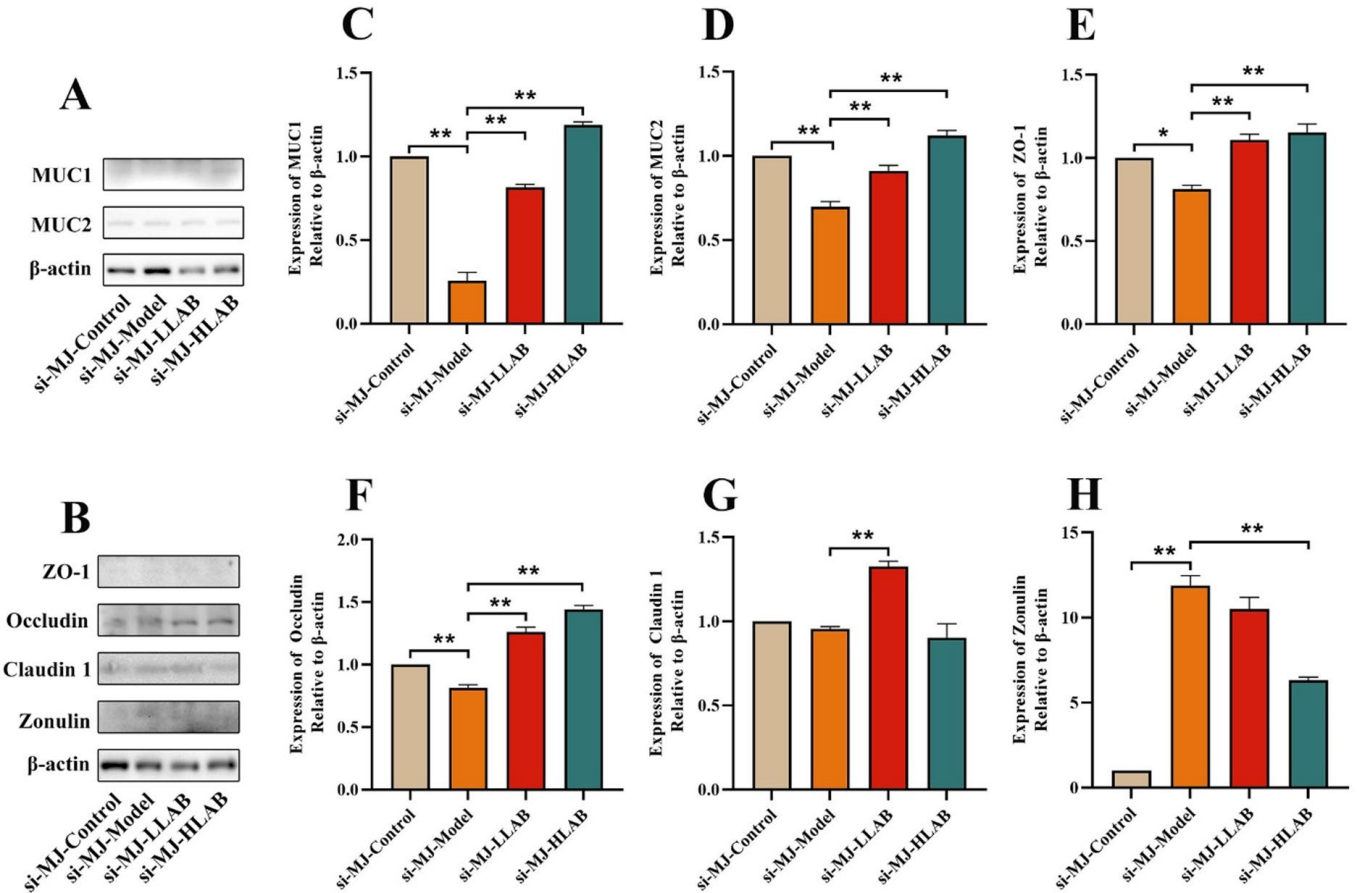
The expression levels of mucin and TJ protein in each treatment group after Zonulin knockdown cell line was supplemented with MAPK activator. (**A**) Gray value detection results of mucin WB; (**B**) Gray value of TJ protein WB; (**C**) Relative β-actin expression level of MUC1; (**D**) Relative β-actin expression level of MUC2; (**E**) Relative β-actin expression level of ZO-1; (**F**) Relative β-actin expression level of Occludin; (**G**) Claudin 1 relative β-actin expression level; (**H**) Relative β-actin expression levels of Zonulin. *p < 0.05, **p < 0.01.

Figure 14 shows the Western bloting results of mucin and TJ proteins in each group of intestinal epithelial cells treated with MAPK activator in the Zonulin knockdown intestinal epithelial cell line. The results showed that the protein expression levels of MUC1, MUC2 and Occludin in si-MJ-Model were significantly decreased (*p* < 0.01), ZO-1 was significantly decreased (*p* < 0.05), and Zonulin was significantly increased (*p* < 0.01). The aberrant expression of these proteins was restored to a certain extent after the addition of *Lactobacillus yoelii* Lac-2. MUC1, MUC2, ZO-1, Occludin, and Claudin 1 with low-dose *Lactobacillus yoelii* Lac-2 were significantly restored (*p* < 0.01). MUC1, MUC2, ZO-1, Occludin, and Zonulin were significantly restored with high doses of *Lactobacillus yoelii* Lac-2 (*p* < 0.01).

### Detection results of protein expression levels of mucin and TJ protein in intestinal epithelial cells with MAPK blocker added to Zonulin knockdown cell line

**Fig. 15:**
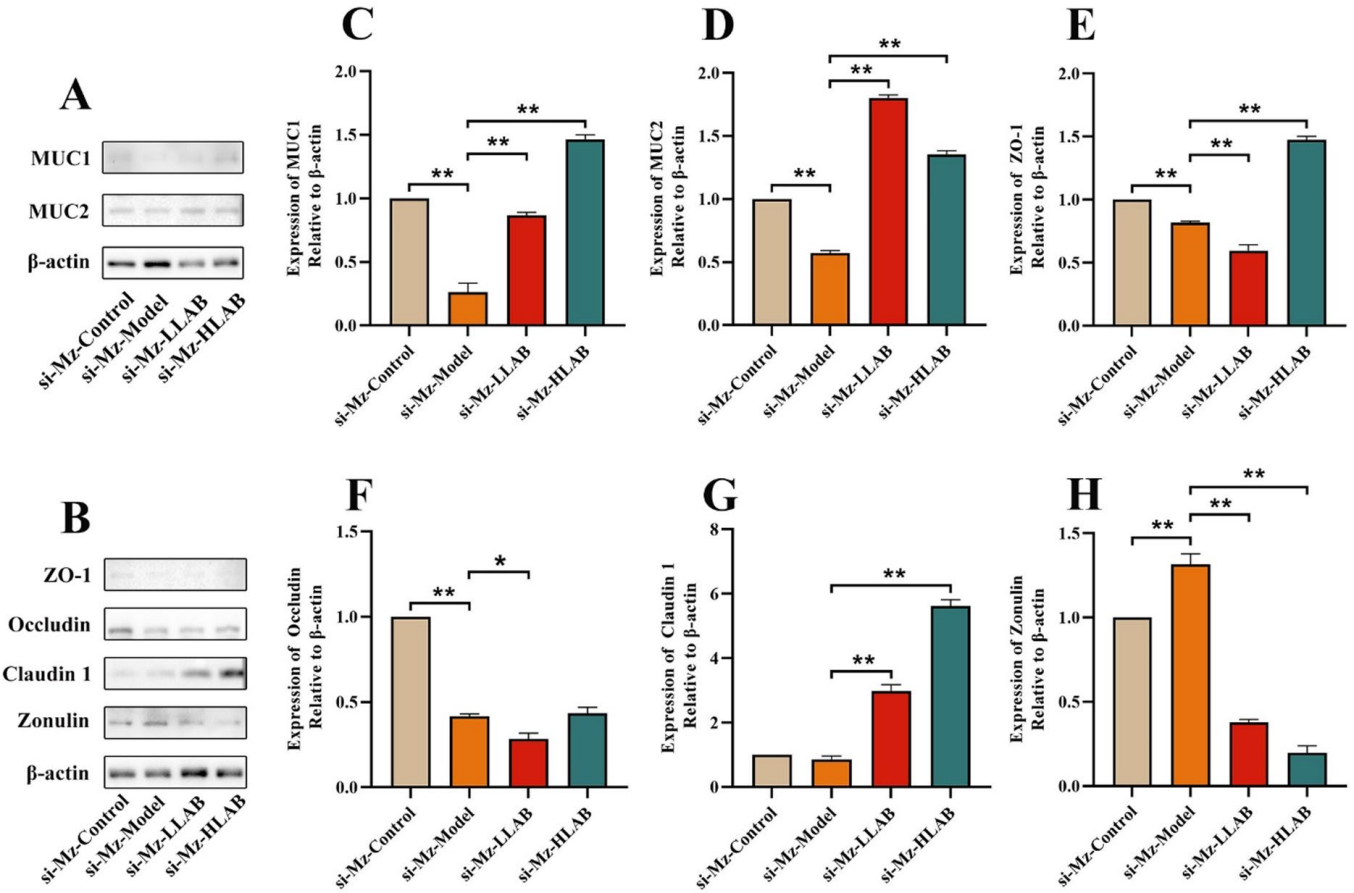
The expression levels of mucin and TJ protein in each treatment group after Zonulin knockdown cell line was supplemented with MAPK blocker. (**A**) Gray value detection results of mucin WB; (**B**) Gray value of TJ protein WB; (**C**) Relative β-actin expression level of MUC1; (**D**) Relative β-actin expression level of MUC2; (**E**) Relative β-actin expression level of ZO-1; (**F**) Relative β-actin expression level of Occludin; (**G**) Claudin 1 relative β-actin expression level; (**H**) Relative β-actin expression levels of Zonulin. *p < 0.05, **p < 0.01.

Figure 15 shows the Western bloting results of mucin and TJ proteins in each group of intestinal epithelial cells treated with MAPK blocker in the Zonulin knockdown intestinal epithelial cell line. The results showed that the protein expression levels of MUC1, MUC2, ZO-1 and Occludin of si-Mz-Model were significantly decreased compared with those of si-Mz-Control (*p* < 0.01), and Zonulin was significantly increased (*p* < 0.01). The aberrant expression of these proteins was restored to a certain extent after the addition of *Lactobacillus yoelii* Lac-2. MUC1, MUC2, Claudin 1, and Zonulin were significantly restored with low-dose *Lactobacillus yoelii* Lac-2 (*p* < 0.01). MUC1, MUC2, ZO-1, Claudin 1, and Zonulin were significantly restored with high doses of *Lactobacillus yoelii* Lac-2 (*p* < 0.01).

### Detection of protein expression levels of mucin and TJ protein in intestinal epithelial cells of HLAB group with MAPK activation and MAPK blocker in Zonulin up-regulation and Zonulin knockdown cell lines

**Fig. 16:**
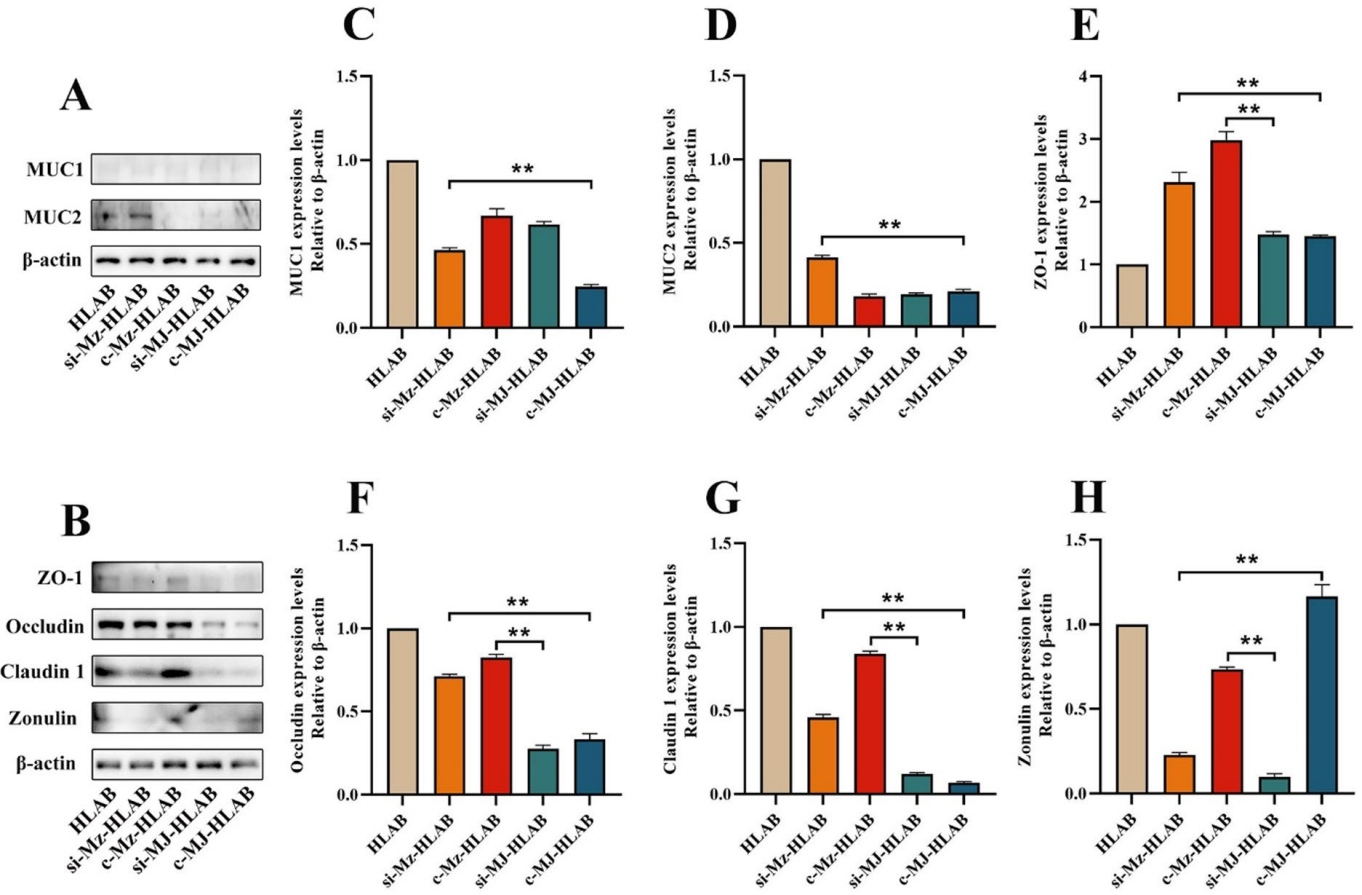
Zonulin up-regulation and Zonulin knockdown cell lines were treated with MAPK activator and MAPK blocker. (**A**) Gray value detection results of mucin WB; (**B**) Gray value of TJ protein WB; (**C**) Relative β-actin expression level of MUC1; (**D**) Relative β-actin expression level of MUC2; (**E**) Relative β-actin expression level of ZO-1; (**F**) Relative β-actin expression level of Occludin; (**G**) Claudin 1 relative β-actin expression level; (**H**) Relative β-actin expression levels of Zonulin. *p < 0.05, **p < 0.01.

Figure 16 shows Western blot assays of mucin and TJ proteins in intestinal epithelial cells treated with MAPK activator and MAPK blocker in Zonulin-overexpressing and Zonulin-knockdown intestinal epithelial cell lines. The results showed that the protein expression levels of MUC1, MUC2, ZO-1, Occludin, and Claudin 1 in the si-Mz-HLAB group were significantly increased (*p* < 0.01), and the protein expression levels of Zonulin were significantly decreased in the si-Mz-HLAB group (*p* < 0.01), which was consistent with the results of RT-qPCR. Compared with the si-MJ-HLAB group, the protein expression levels of ZO-1, Occludin, Claudin 1 and Zonulin in the c-Mz-HLAB group were significantly increased (*p* < 0.01), which was consistent with the results of RT-qPCR.

### Detection results of intestinal epithelial cell apoptosis of Zonulin up-regulation cell line with MAPK activator

**Fig. 17:**
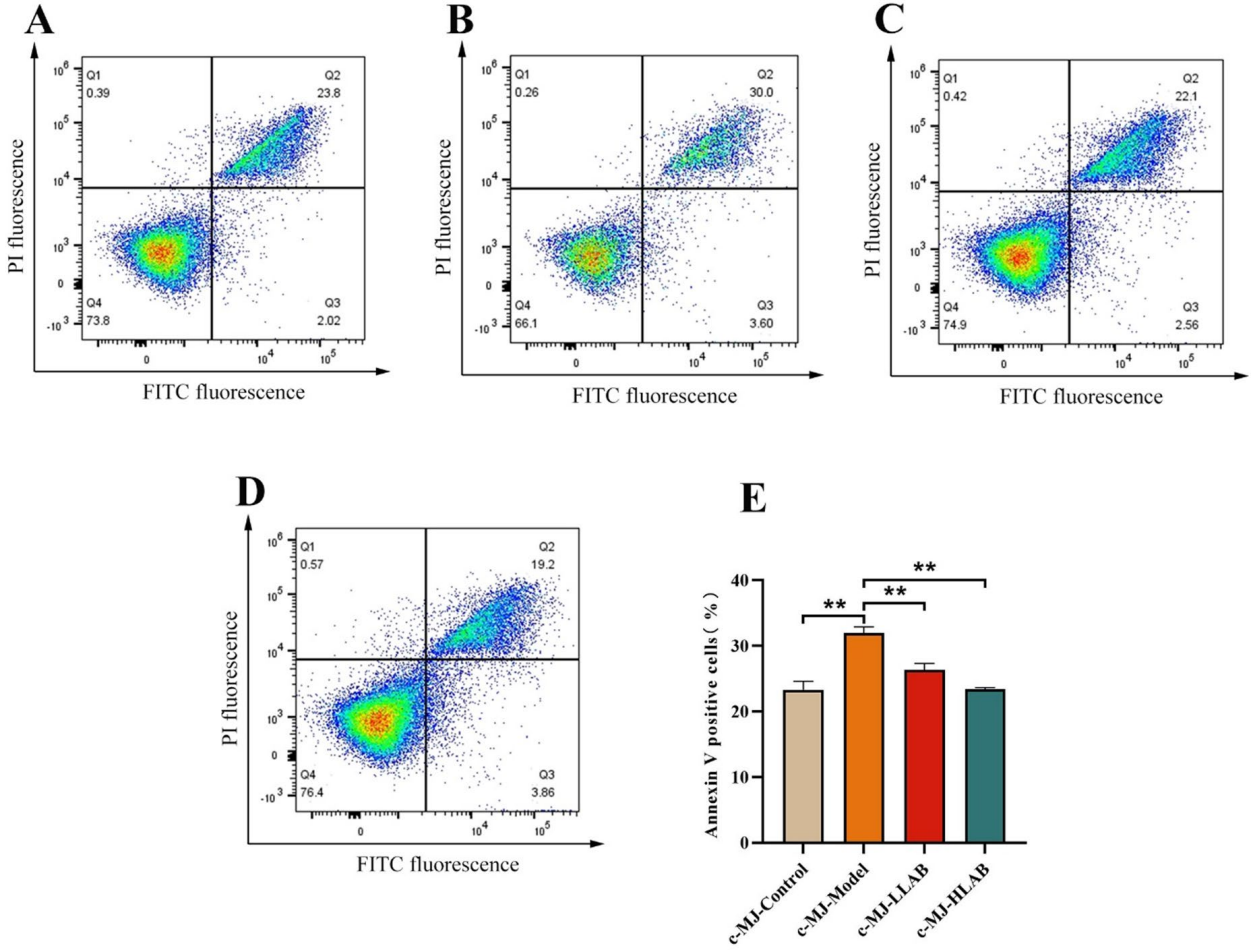
Intestinal epithelial cells in each treatment group after the addition of MAPK activator to the Zonulin up-regulation cell line were double-stained with PI and Annexin V, and analyzed by flow cytometry to assess apoptosis. (**A**) Apoptosis rate in the c-MJ-Control group; (**B**) Apoptosis rate in the c-MJ-Model group; (**C**) Apoptosis rate in the c-MJ-LLAB group; (**D**) Apoptosis rate in the c-MJ-HLAB group; (**E**) Quantification of the percentage of apoptotic cells. *p < 0.05, **p < 0.01.

Figure 17 shows the results of apoptosis assays after treatment with the addition of MAPK activator to the Zonulin-overexpressing intestinal epithelial cell line. Annexin V and PI staining showed that the addition of *E.coli* O78 resulted in a significant increase in the apoptosis rate of intestinal epithelial cells compared with the c-MJ-Control group (*p* < 0.01). Compared with the c-MJ-Model group, the apoptosis rate of intestinal epithelial cells was significantly decreased after the addition of *Lactobacillus yoelii* Lac-2 (*p* < 0.01), and the downward trend was dose-dependent on *Lactobacillus yoelii* Lac-2.

### Zonulin up-regulation cell line with MAPK blocker intestinal epithelial cell apoptosis results

**Fig. 18:**
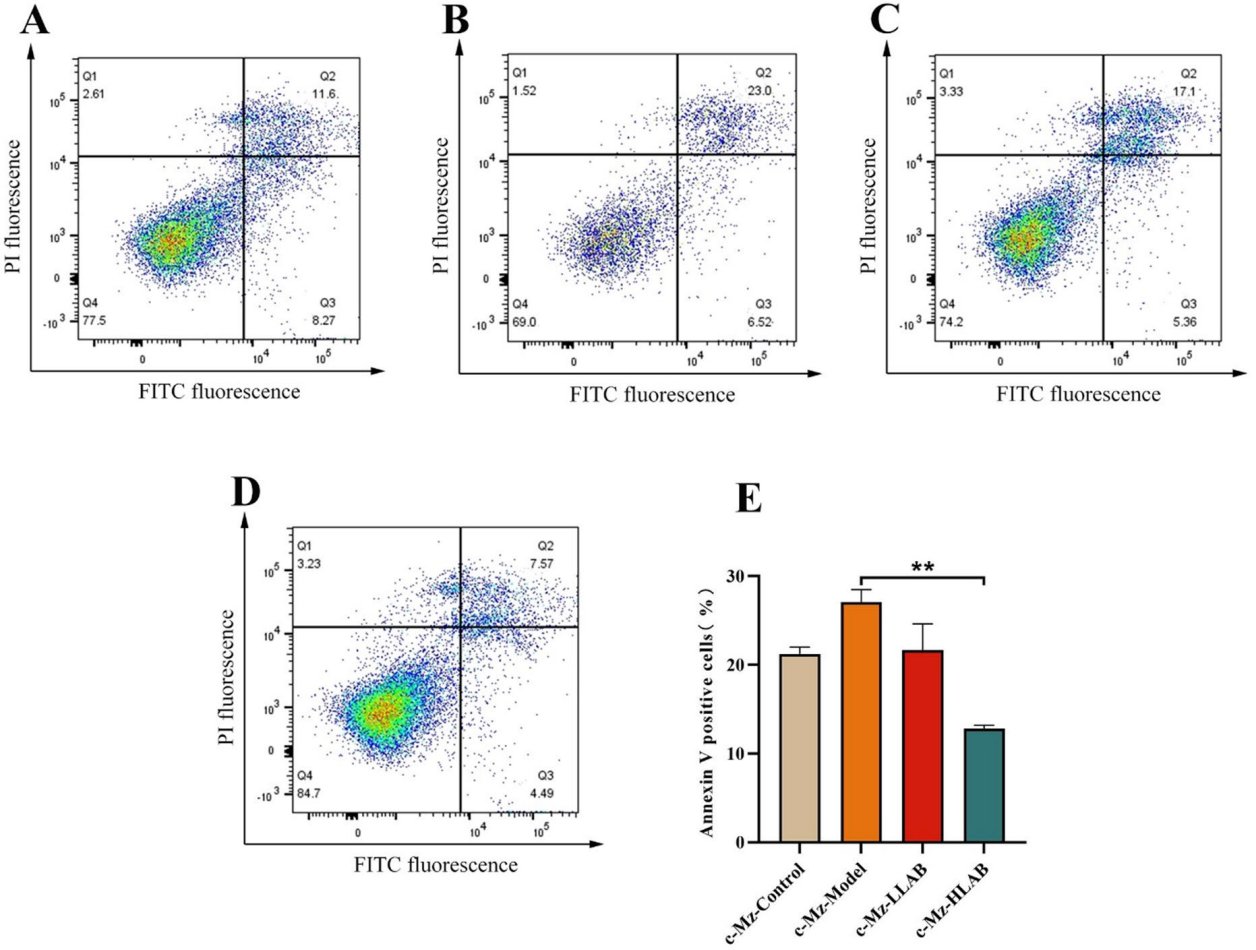
Intestinal epithelial cells in each treatment group of Zonulin overexpressed cell lines after MAPK blockade were double-stained with PI and Annexin V, and analyzed by flow cytometry to assess apoptosis. (**A**) Apoptosis rate in the c-Mz-Control group; (**B**) Apoptosis rate in the c-Mz-Model group; (**C**) Apoptosis rate in the c-Mz-LLAB group; (**D**) Apoptosis rate in the c-Mz-HLAB group; (**E**) Quantification of the percentage of apoptotic cells. *p < 0.05, **p<0.01.

Figure 18 shows the results of apoptosis assays after treatment with the addition of MAPK blockade to the Zonulin-overexpressing intestinal epithelial cell line. Annexin V and PI staining showed that the addition of high doses of *Lactobacillus yoelii* Lac-2 resulted in a significant decrease in the apoptosis rate of intestinal epithelial cells compared with the c-MJ-Model group (*p* < 0.01).

### Detection results of intestinal epithelial cell apoptosis with MAPK activator added to Zonulin knockdown cell line

**Fig. 19:**
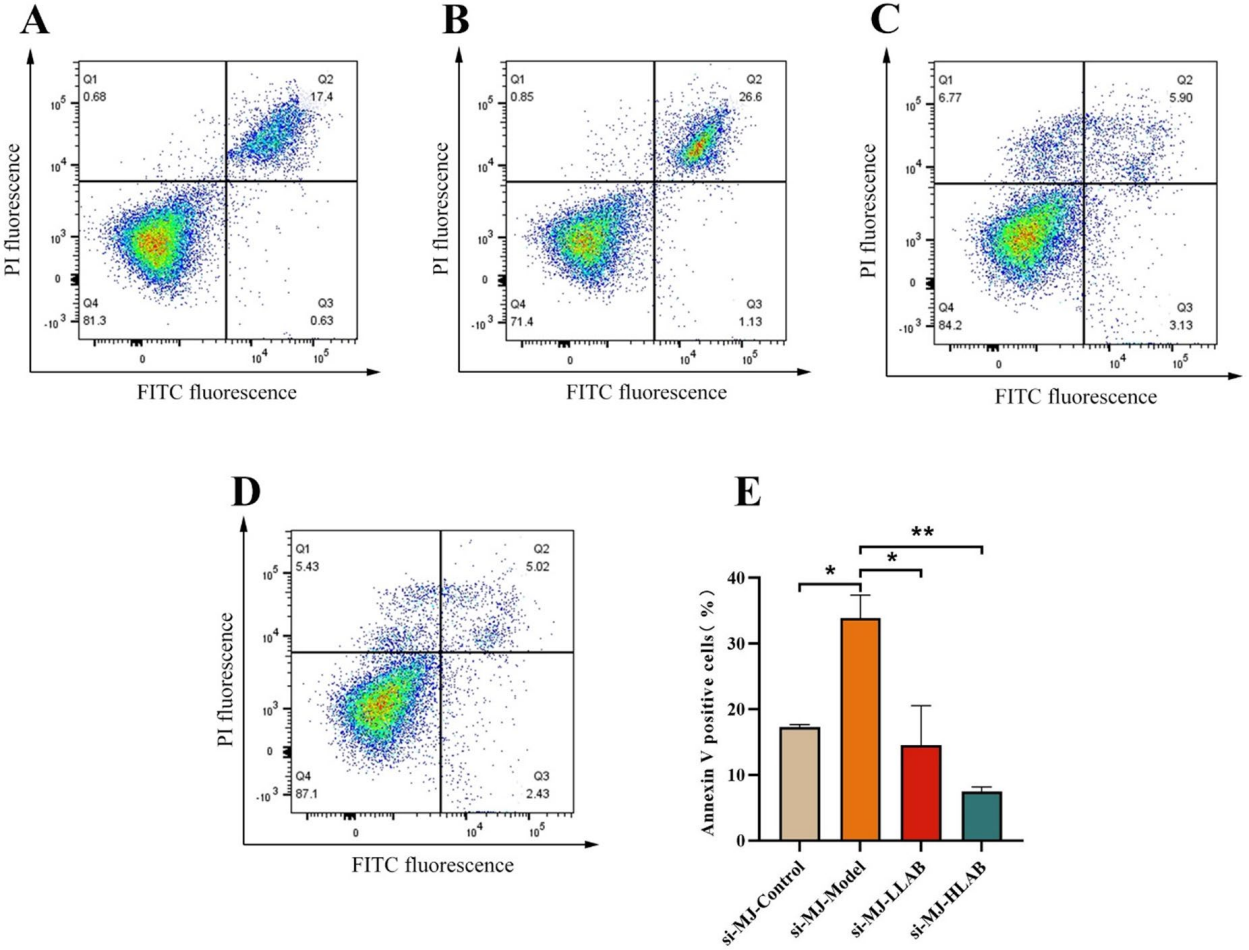
Intestinal epithelial cells in each treatment group after Zonulin knockdown cell line and MAPK activator were double-stained with PI and Annexin V, and analyzed by flow cytometry to assess apoptosis. (**A**) Apoptosis rate in the si-MJ-Control group; (**B**) Apoptosis rate in the si-MJ-Model group; (**C**) Apoptosis rate in the si-MJ-LLAB group; (**D**) Apoptosis rate in the si-MJ-HLAB group; (**E**) Quantification of the percentage of apoptotic cells. *p < 0.05, **p < 0.01.

Figure 19 shows the results of apoptosis assays after treatment with the addition of MAPK activator to the Zonulin knockdown intestinal epithelial cell line. Annexin V and PI staining showed that the addition of *E.coli* O78 resulted in a significant increase in the apoptosis rate of intestinal epithelial cells compared with the si-MJ-Control group (*p* < 0.05). Compared with the si-MJ-Model group, the addition of low dose of *Lactobacillus yoelii* Lac-2 resulted in a significant decrease in the apoptosis rate of intestinal epithelial cells (*p* < 0.05), and the addition of high dose of *Lactobacillus yoelii* Lac-2 resulted in a significant decrease in the apoptosis rate of intestinal epithelial cells (*p* < 0.01).

### Zonulin knockdown cell line with MAPK blocker intestinal epithelial cell apoptosis test results

**Fig. 20:**
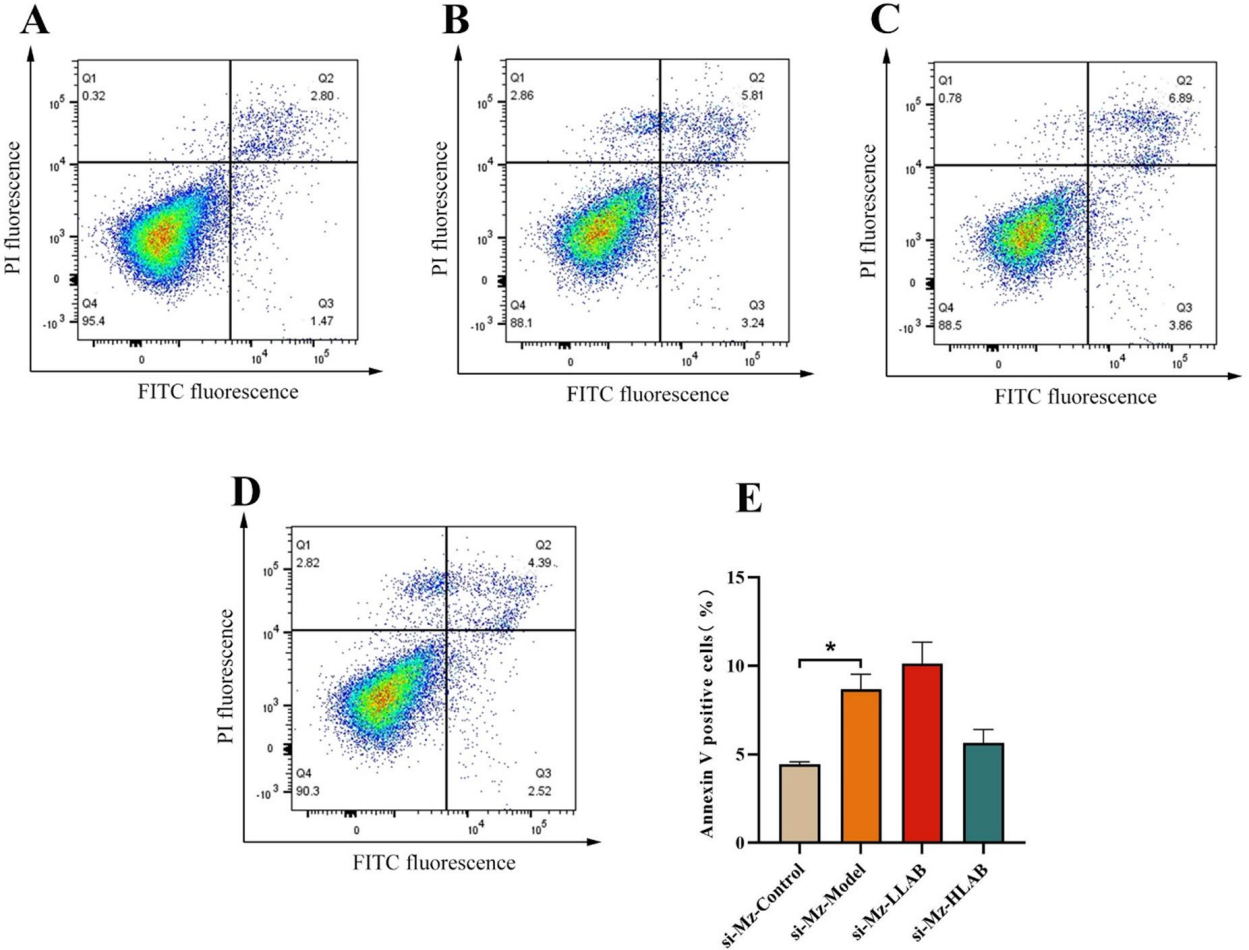
Intestinal epithelial cells in each treatment group after Zonulin knockdown cell line and MAPK blockade were double-stained with PI and Annexin V, and analyzed by flow cytometry to assess apoptosis. (**A**) Apoptosis rate in the si-Mz-Control group; (**B**) Apoptosis rate in the si-Mz-Model group; (**C**) Apoptosis rate in the si-Mz-LLAB group; (**D**) Apoptosis rate in the si-Mz-HLAB group; (**E**) Quantification of the percentage of apoptotic cells. *p < 0.05, **p < 0.01.

Figure 20 shows the results of apoptosis assays after treatment with the addition of MAPK blockade in the Zonulin knockdown intestinal epithelial cell line. Annexin V and PI staining showed that the addition of *E.coli* O78 resulted in a significant increase in the apoptosis rate of intestinal epithelial cells compared with the si-Mz-Control group (*p* < 0.01).

### Apoptosis in the HLAB group of Zonulin up-regulation and Zonulin knockdown cell lines with MAPK activation and MAPK blocker

**Fig. 21:**
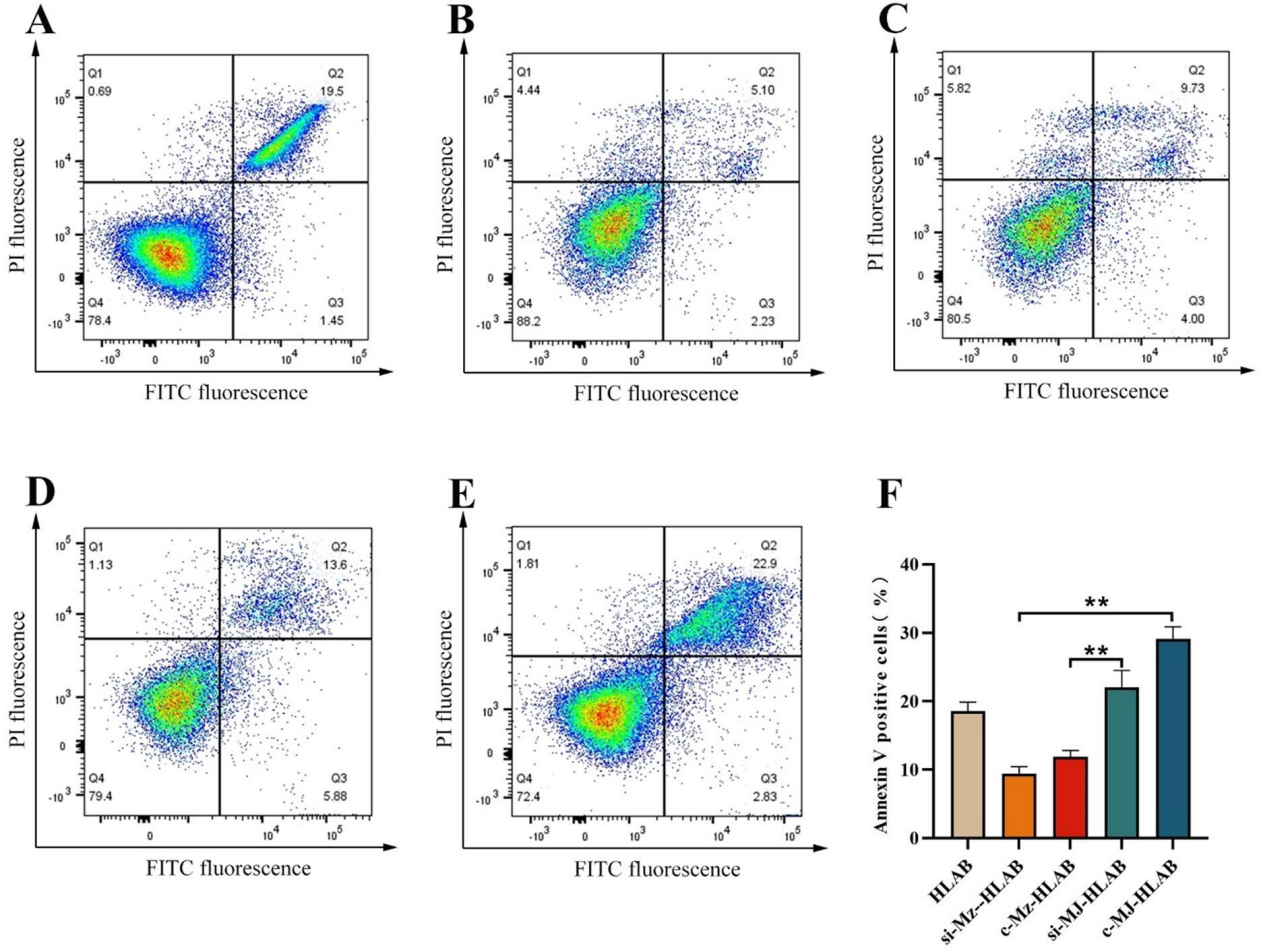
Intestinal epithelial cells in the HLAB group of Zonulin up-regulation and Zonulin knockdown cell lines after the addition of MAPK activator and MAPK blocker were double-stained with PI and Annexin V, and analyzed by flow cytometry to assess apoptosis. (**A**) Apoptosis rate in the HLAB group; (**B**) Apoptosis rate in the si-Mz-HLAB group; (**C**) Apoptosis rate in the c-Mz-HLAB group; (**D**) Apoptosis rate in the si-MJ-HLAB group; (**E**) Apoptosis rate in the c-MJ-HLAB group; (**F**) Quantification of the percentage of apoptotic cells. *p < 0.05, **p < 0.01.

Figure 21 shows the results of apoptosis assays after treatment with MAPK activator and MAPK blocker in Zonulin up-regulation and Zonulin knockdown intestinal epithelial cell lines. Annexin V and PI staining showed that the apoptosis rate of the si-Mz-HLAB group was significantly lower than that of the c-MJ-HLAB group (*p* < 0.01), and the apoptosis rate of the c-Mz-HLAB group was significantly lower than that of the si-MJ-HLAB group (*p* < 0.01).

## Discussion

*LAB* is a common intestinal probiotic, and probiotics have been shown to alter dysregulated gut microbiota^27^. In this trial, we investigated Zonulin up-regulation and Zonulin knockdown intestinal epithelial cell lines. Transwell inserts are cultured with intestinal epithelial cell lines to construct a monolayer of epithelial cell barriers. MAPK activator and MAPK blocker were added to the above treatment groups, and then the mechanism of yak-derived *Lactobacillus yoelii* Lac-2 mediated MAPK pathway in regulating the intestinal barrier and pathogen translocation of Zonulin expression by *Lactobacillus yoelii* was explored through the four experimental groups: Control group, Model group, LLAB group and HLAB group. Our results found that both up-regulation of Zonulin and activation of the MAPK pathway exacerbated the damage of *E.coli* O78 to the intestinal epithelial cell barrier and decreased the protective effect of yak-derived *Lactobacillus yoelii* Lac-2. Up-regulation of Zonulin and activation of the MAPK pathway further exacerbated the damage of *E.coli* O78 to the intestinal epithelial cell barrier, and further reduced the protective effect of yak-derived *Lactobacillus yoelii* Lac-2. Knockdown of Zonulin or blockade of the MAPK pathway reduced the damage of *E.coli* O78 to the intestinal epithelial cell barrier and enhanced the protective effect of yak-derived *Lactobacillus yoelii* Lac-2. Knockdown of Zonulin and blockade of the MAPK pathway at the same time will make the effect more pronounced. This suggests that the protective mechanism of yak-derived *Lactobacillus yoelii* Lac-2 against yak-derived *E.coli* O78-induced damage to the yak intestinal epithelial barrier is: Yak-derived *Lactobacillus yoelii* Lac-2 maintains the integrity of the intestinal epithelial barrier by blocking the MAPK pathway, which in turn reduces the production of Zonulin, which in turn reduces the opening of tight junctions, reduces pathogenic translocations, and maintains the integrity of the intestinal epithelial barrier.

Elevated serum levels of Zonulin, a factor that regulates intestinal tight junctions, are associated with increased intestinal permeability, dyspepsia, and inflammation^28^. The MAPK family includes ERK1/2, JNK1-3, p38, and ERK5, which regulate protein levels and function. Studies have shown that intestinal PRR recognizes bacterial components to activate MAPK/NF-κB, promote cytokine release and innate immunity, and eliminate pathogens; Bacteria, on the other hand, use effector proteins and toxins to intervene in MAPK signaling and immune responses^29,30^. The mRNA and protein expression levels of p38, ERK, JNK, and Zonulin were detected by constructing Zonulin up-regulation and Zonulin knockdown cell lines, and then adding MAPK activator and MAPK blocker, respectively, using RT-qPCR and Western blot technology. The results showed that the mRNA and protein expression levels of p38, ERK, JNK, and Zonulin in the si-MJ-Control group, si-Mz-Control group, and c-Mz-Control group were significantly reduced compared with the control group (*p* < 0.01), and the mRNA levels of p38, ERK, JNK, and Zonulin in the c-MJ-Control group were significantly reduced and protein expression levels were significantly increased (*p* < 0.01). This result indicates that the construction of Zonulin up-regulation and Zonulin knockout cell lines was successful, as was the addition of MAPK activator and MAPK blocker, respectively. Compromised intestinal barriers often result in increased permeability, bacterial translocation, and increased FD4 flux levels^31^. In this trial, we simulated E. coli’s damage to the intestinal barrier by adding 200 µL of 1 × 10^5^ CFU/mL of *E.coli* O78 to the constructed model. Different doses of yak-derived *Lactobacillus yoelii* Lac-2 (200 µL 1 × 10^5^ CFU/mL and 200 µL 1 × 10^7^ CFU/mL, respectively) were added to simulate the effects of different doses of probiotics on the intestinal barrier. The results showed that compared with the control group, the transmembrane resistance within 4 h and the transmembrane resistance value at 4 h in the experimental group with only *E.coli* O78 showed a decreasing trend relative to the level percentage of the control group, while the bacterial translocation and FD4 flux within 4 h both showed an upward trend. Compared with the experimental group with *E.coli* O78 only, we found that the transmembrane resistance within 4 h and the transmembrane resistance at 4 h in the experimental group supplemented with *Lactobacillus yoelii* Lac-2 increased by percentage relative to the control group, while the bacterial translocation and FD4 flux decreased within 4 h. At the same time, we also compared the si-Mz-HLAB group with the c-MJ-HLAB group and found that the transmembrane resistance of the si-Mz-HLAB group was significantly higher than that of the c-MJ-HLAB group, the percentage of transmembrane resistance at 4 h in the si-Mz-HLAB group was significantly higher than that in the c-MJ-HLAB group (*p* < 0.01), and the bacterial translocation in the si-Mz-HLAB group was significantly lower than that in the c-MJ-HLAB group within 4 h. The FD4 flux of the si-Mz-HLAB group was significantly lower than that of the c-MJ-HLAB group. These results suggest that yak-derived *Lactobacillus yoelii* Lac-2 has a protective effect on the intestinal barrier by blocking the MAPK pathway, reducing the amount of bacterial translocation, and reducing the expression of Zonulin. Subsequently, we compared the si-MJ-HLAB group with the c-Mz-HLAB group, and found that the transmembrane resistance of the si-MJ-HLAB group was slightly lower than that of the c-Mz-HLAB group within 1–4 h, the percentage of transmembrane resistance relative to the HLAB group at 4 h was significantly lower than that of the c-Mz-HLAB group, the bacterial translocation within 4 h was slightly higher than that of the c-Mz-HLAB group, and the FD4 flux was significantly higher than that of the c-Mz-HLAB group. These results indicated that the protective effect of *Lactobacillus yoelii* Lac-2 from yak was achieved by blocking the MAPK signaling pathway, thereby reducing the amount of bacterial translocation and reducing the expression of Zonulin.

The intestinal tight junction (TJ) barrier is essential to prevent intestinal contents from penetrating into the intestinal wall, tissues, and blood circulation^32^. The TJ complex consists of occlusin (Occludin), claudin protein, and accessory proteins (e.g., ZO-1, ZO-2, and ZO-3)^33–35^. These TJs are essential for maintaining epithelial cell polarity and cell bypass barrier function, and they form a physical line of defense against microorganisms, antigens, and foreign bodies^36^. Tight junction proteins are important components of the epithelial barrier, and damage to the epithelial barrier can lead to disturbed expression of tight junction proteins^26^. The MUC1 and MUC2 mucins secreted by goblet cells play a key role in defending against microbial invasion and attenuating the subsequent inflammatory response^37^. In this trial, we compared the experimental group with only *E.coli* O78 with the experimental group without the addition of bacterial solution. It was found that the mRNA and protein expressions of MUC1, MUC2, ZO-1, Occludin, Claudin 1 and Zonulin were disordered, while the mRNA and protein expression levels of MUC1, MUC2, ZO-1, Occludin, Claudin 1 and Zonulin were restored to a certain extent in the experimental group with yak-derived *Lactobacillus yoelii* Lac-2. The results of our trial are almost consistent with those of Zhang et al.^26^. In our trial, we also compared the mRNA and protein expression levels of MUC1, MUC2, ZO-1, Occludin, and Claudin 1 in the si-Mz-HLAB group with the c-MJ-HLAB group (*p* < 0.01), and the mRNA and protein expression levels of Zonulin were significantly higher than those in the c-MJ-HLAB group (*p* < 0.01). These results indicated that the protective effect of *Lactobacillus yoelii* Lac-2 from yak was achieved by blocking the MAPK pathway and reducing the expression of Zonulin. We further compared the mRNA and protein expression levels of ZO-1, Occludin, and Claudin 1 in the c-MZ-HLAB group with the c-Mz-HLAB group and found that the mRNA and protein expression levels of ZO-1, Occludin, and Claudin 1 in the c-Mz-HLAB group were significantly lower than those in the si-MJ-HLAB group. This further indicates that the protective effect of yak-derived *Lactobacillus yoelii* Lac-2 on the intestinal epithelial cell barrier is achieved by blocking the MAPK pathway, thereby reducing the expression of Zonulin.

A growing body of research supports the idea that abnormally increased mortality of intestinal epithelial cells (IECs) through apoptosis, pyroptosis, or necroptosis is directly related to the immune response and barrier integrity of the intestinal mucosa^38–40^. By comparing the apoptosis rate of *E.coli* O78 with that of the non-administered group, we found that *E.coli* O78 increased the apoptosis rate of intestinal epithelial cells in both overexpressing and knocking down Zonulin cell lines. Subsequently, we compared the experimental group with *E.coli* O78 and different doses of yak-derived *Lactobacillus yoelii* Lac-2 with *E.coli* O78 alone, and found that the apoptosis rate was reduced in the experimental group with low doses of *Lactobacillus yoelii* Lac-2 from yak, and even more in the experimental group with high doses of *Lactobacillus yoelii* Lac-2 from yak. These results indicated that *E.coli* O78 could increase the apoptosis rate of intestinal epithelial cells in both overexpressing and knocking down Zonulin cell lines, while *Lactobacillus yoelii* Lac-2 from yak could reduce intestinal epithelial cell apoptosis. This is in line with the point we mentioned earlier. We further compared the HLAB group that knocked down Zonulin and added MAPK blocker to the HLAB group that overexpressed Zonulin and added MAP blocker. It was found that the apoptosis rate was significantly lower in the HLAB group that knocked down Zonulin and added MAPK blocker than in the HLAB group that overexpressed Zonulin and added MAPK blocker. These results suggest that *Lactobacillus yoelii* Lac-2 of yak origin can effectively reduce the apoptosis of intestinal epithelial cells by blocking the MAPK pathway and reducing the expression of Zonulin. We then compared the HLAB group that knocked down Zonulin and added the MAPK activator to the HLAB group that overexpressed Zonulin and blocked the MAPK pathway. It was found that the HLAB group that knocked down Zonulin and added the MAPK activator was significantly higher than the HLAB group that overexpressed Zonulin and blocked the MAPK pathway. These results indicate that the protective effect of yak-derived *Lactobacillus yoelii* Lac-2 on intestinal epithelial cells is achieved by first blocking the MAPK pathway and then reducing the expression of Zonulin. This has been found to echo the results of our previous trials.

Finally, we concluded that yak-derived *Lactobacillus yoelii* Lac-2 protects against the damage of the intestinal epithelial cell barrier caused by *E.coli* O78 by blocking the MAPK pathway, reducing pathogen translocation, increasing the expression of mucin (MUC1, MUC2) and TJ proteins (ZO-1, Occludin, Claudin 1), and reducing the production of Zonulin.

## Conclusion

This study systematically evaluated the protective effect and underlying mechanism of yak-derived *Lactobacillus yoelii* Lac-2 against *Escherichia coli* O78-induced intestinal epithelial barrier damage in yaks. Our results demonstrated that *E. coli* O78 stimulation significantly activated the MAPK signaling pathway and upregulated Zonulin expression in yak intestinal epithelial cells, which subsequently downregulated the mRNA and protein levels of tight junction proteins (ZO-1, Occludin, Claudin-1) and mucins (MUC1, MUC2), reduced transepithelial electrical resistance (TEER), increased FITC-dextran (FD4) permeability and bacterial translocation, and promoted cell apoptosis. Notably, treatment with *Lactobacillus yoelii* Lac-2 effectively reversed these adverse effects by inhibiting MAPK pathway activation and reducing Zonulin production, thereby maintaining the integrity of the intestinal epithelial barrier. Despite these findings, the present study has inherent limitations: all experiments were conducted using a monoculture intestinal epithelial cell model, which fails to replicate the complex in vivo intestinal microenvironment, including microbial community interactions and systemic immune responses; additionally, only one cell line and a single detection time point were used, which may limit the generalizability of our results. Future research should extend these observations to in vivo yak models to validate the protective efficacy of *Lactobacillus yoelii* Lac-2 in experimental or natural diarrhea. Furthermore, optimizing the dosage and administration route of *Lactobacillus yoelii* Lac-2, exploring its synergistic effects with other probiotics, and investigating the crosstalk between the MAPK pathway and other intestinal barrier-related signaling cascades will help deepen our understanding of its mechanism of action and facilitate its practical application in yak husbandry as an alternative to antibiotics.

## Supplementary Information

Below is the link to the electronic supplementary material.

Supplementary Material 1

## Data Availability

All data generated or analysed during this study are included in this published article.
