# Supplementary material for: Lactobacillus yoelii Lac-2 mediates the mechanism of MAPK signaling pathway involved in the regulation of intestinal barrier and pathogen translocation by Zonulin expression
Source: Sci Rep. 2026 Jun 3;16:25256. doi: 10.1038/s41598-026-55430-y (PMC13469141; doi:10.1038/s41598-026-55430-y)
Supplement: Supplementary file 1 [file 41598_2026_55430_MOESM1_ESM.docx]

Figure 12 A

|  | MUC1 |
| --- | --- |
|  |  |
|  | MUC2 |
|  |  |
|  | β-actin |
|  |  |

Figure 12 B

|  | ZO-1 |
| --- | --- |
|  |  |
|  | Occludin |
|  |  |
|  | Claudin1 |
|  |  |
|  | Zonulin |
|  |  |
|  | β-actin |
|  |  |

Figure 13 A

|  | MUC1 |
| --- | --- |
|  |  |
|  | MUC2 |
|  |  |
|  | β-actin |
|  |  |

Figure 13 B

|  | ZO-1 |
| --- | --- |
|  |  |
|  | Occludin |
|  |  |
|  | Claudin1 |
|  |  |
|  | Zonulin |
|  |  |
|  | β-actin |
|  |  |

Figure 14 A

|  | MUC1 |
| --- | --- |
|  |  |
|  | MUC2 |
|  |  |
|  | β-actin |
|  |  |

Figure 14 B

|  | ZO-1 |
| --- | --- |
|  |  |
|  | Occludin |
|  |  |
|  | Claudin1 |
|  |  |
|  | Zonulin |
|  |  |
|  | β-actin |
|  |  |

Figure 15 A

|  | MUC1 |
| --- | --- |
|  |  |
|  | MUC2 |
|  |  |
|  | β-actin |
|  |  |

Figure 15 B

|  | ZO-1 |
| --- | --- |
|  |  |
|  | Occludin |
|  |  |
|  | Claudin1 |
|  |  |
|  | Zonulin |
|  |  |
|  | β-actin |
|  |  |

Figure 16 A

|  | MUC1 |
| --- | --- |
|  |  |
|  | MUC2 |
|  |  |
|  | β-actin |
|  |  |

Figure 16 B

|  | ZO-1 |
| --- | --- |
|  |  |
|  | Occludin |
|  |  |
|  | Claudin1 |
|  |  |
|  | Zonulin |
|  |  |
|  | β-actin |
|  |  |
